# Supplementary material for: A supramolecular system that strictly follows the binding mechanism of conformational selection
Source: Nat Commun. 2020 Jun 2;11:2740. doi: 10.1038/s41467-020-16534-9 (PMC7265396; doi:10.1038/s41467-020-16534-9)
Supplement: Supplementary file 1 — Supplementary Information [file 41467_2020_16534_MOESM1_ESM.pdf]

Supplementary Information

A Supramolecular System That Strictly Follows the Binding Mechanism of Conformational Selection

Liu-Pan Yang,[a] Li Zhang,[a] Mao Quan,[a] Jas S. Ward,[b] Yan-Long Ma,[a] Hang Zhou,[a] Kari Rissanen,[b] and Wei Jiang*[a]

[a] Shenzhen Grubbs Institute, Department of Chemistry, Guangdong Provincial Key Laboratory of Catalysis and Academy for Advanced Interdisciplinary Studies, Southern University of Science and Technology, Xueyuan Blvd 1088, Shenzhen, 518055, China.

[b] University of Jyvaskyla, Department of Chemistry, P. O. Box 35, FI-40014, Jyväskylä, Finland.

*E-mail: jiangw@sustech.edu.cn

Table of Contents

1. Supplementary Figures S3

1.1. Properties of **1** S3
1.2. Guest Binding of Host **1** to Dinitrobenzene **2** S7
1.3. Kinetic Experiments S12
1.4. Binding Constants Determination S14

2. Supplementary Note **1** S27

3. Supplementary Methods S32

3.1. General method S32
3.2. Synthetic Procedures S33
3.3. X-ray Crystallography S44

4. Supplementary References S46

1. Supplementary Figures

1.1. Properties of 1

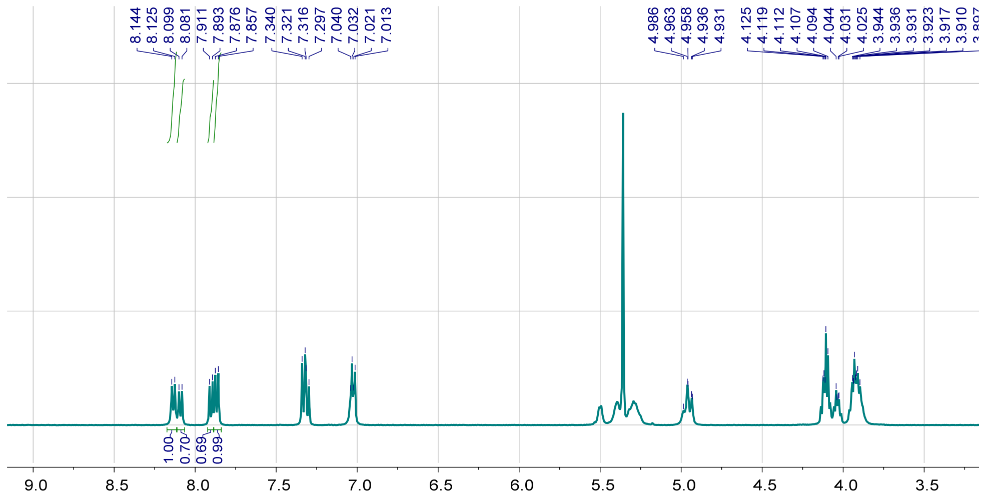

Supplementary Figure 1 Partial ^{1}H NMR spectra (500 MHz, CD_2Cl_2, 2.0 mM, 298 K) of **1**. Integration indicates that there are two sets peaks (2D NMR spectra also supports this, see **Supplementary Figure 2**), each with four doublets. Thus conformers **I** and **V** coexist in the solution.

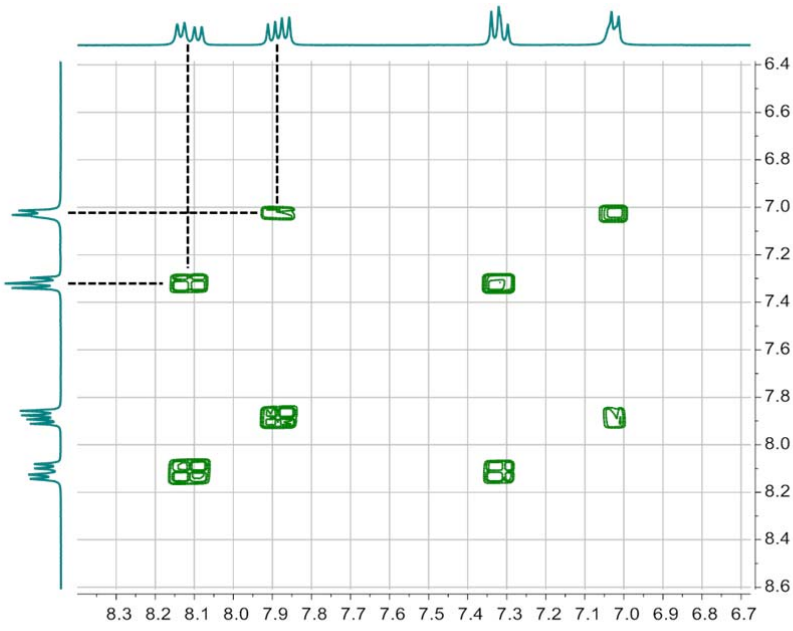

Supplementary Figure 2 Partial ^{1}H-^{1}H COSY spectra (500 MHz, CD_2Cl_2, 6.0 mM, 298 K) of **1**.

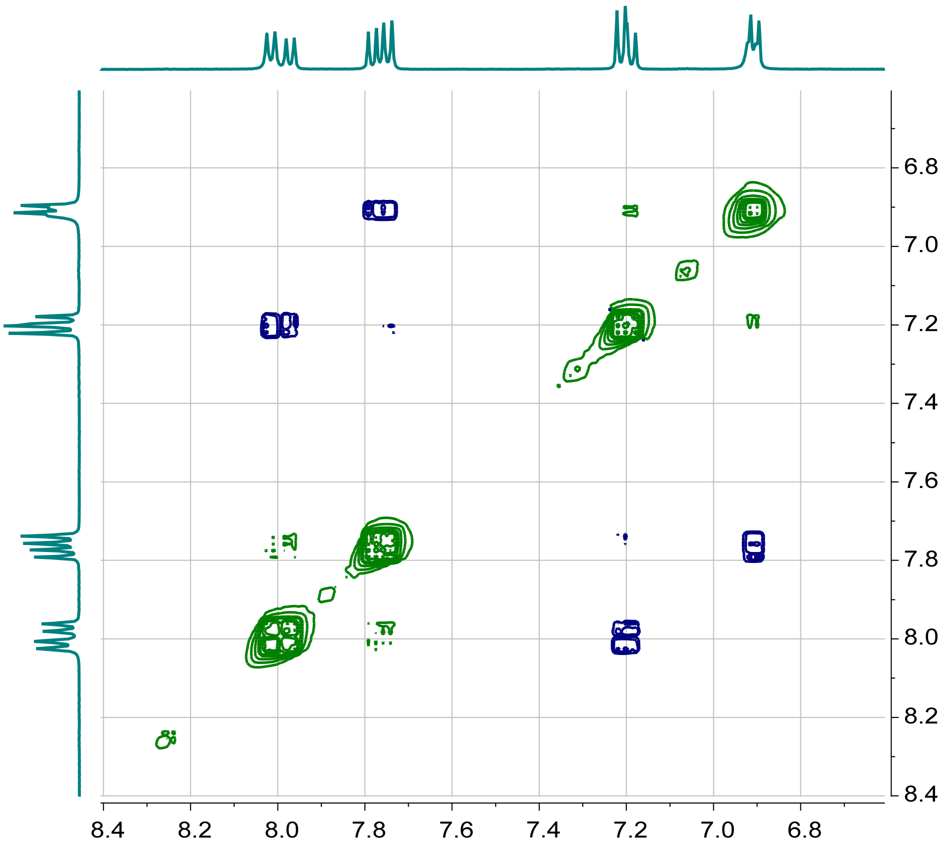

Supplementary Figure 3 Partial ^{1}H-^{1}H ROESY spectra (500 MHz, CD_2Cl_2, 6.0 mM, 298 K) of **1**.

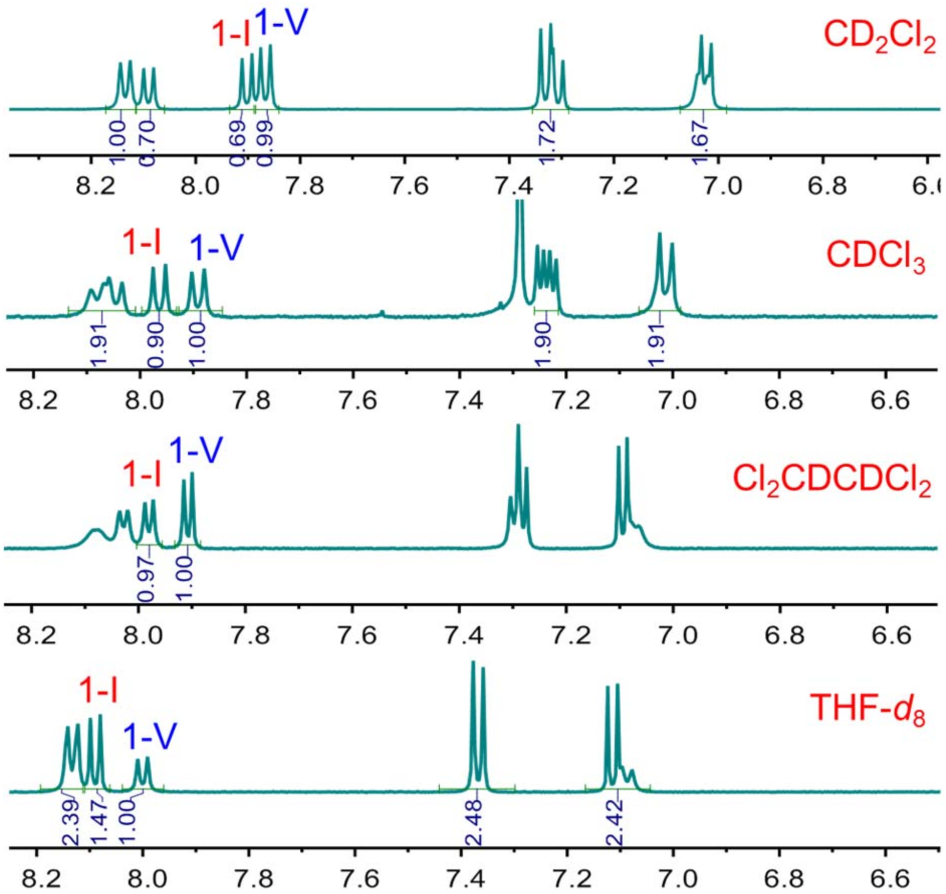

Supplementary Figure 4 Partial ^{1}H NMR spectra (500 MHz, 1.0 mM, 25 °C) of **1** in different deuterated solvents. Conformer **I** and the conformer **V** coexist in all these deuterated solvents, the integral values indicate that the ratio of two conformers in different solvent are slightly different ([**I**]/[**V**] are 0.70, 0.90, 0.97 and 1.47 for in CD_2Cl_2, $CDCl_3$, $Cl_2CDCDCl_2$ and THF-d_8, respectively). The conformations were assigned with the help of titrating 1,4-dinitrobenzene (see ***Supplementary Figures 7***, ***11-13***). No ^{1}H NMR signal can be observed in toluene-d_8, CD_3CN, acetone-d_6 and DMSO-d_6 because of very poor solubility of macrocycle **1** in these solvents. In addition, the splitting of ^{1}H NMR signal of macrocycle **1** in aromatic region is clearer in CD_2Cl_2 than in other deuterated solvents, so the experiments for host-guest chemistry are mainly performed in CD_2Cl_2.

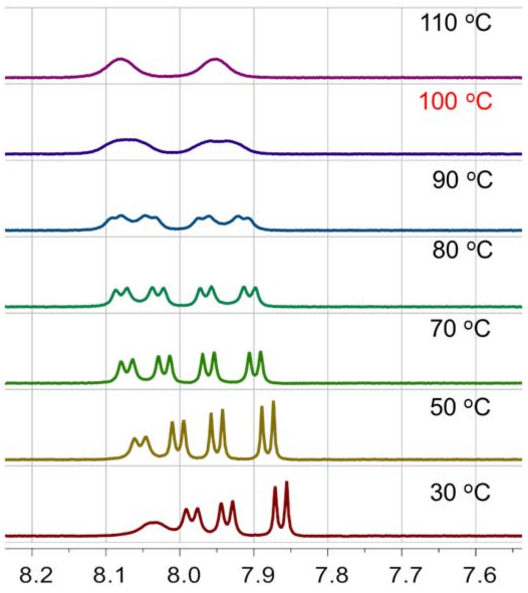

Supplementary Figure 5 Variable-temperature ^{1}H NMR spectra (600 MHz, $Cl_2CDCDCl_2$) of **1**. The $\Delta G^{\ddagger}$ value was estimated by using the coalescence temperatures and the chemical shifts at 30 ^{o}C. The coalescence was reached at ca. 100 ^{o}C. The $\Delta G^{\ddagger}$ value was calculated to be 74 kJ/mol according to the following equation[1]: $\Delta G^{\ddagger} = 8.314T_c[22.96 + \log(T_c/\delta v)]$.

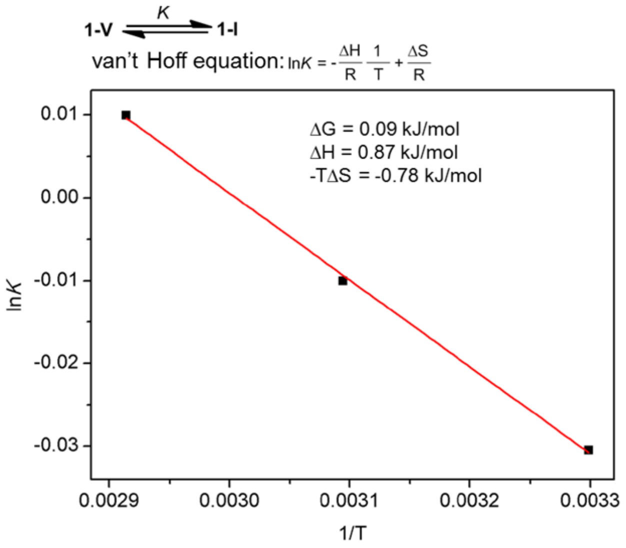

Supplementary Figure 6 van't Hoff equation and van't Hoff plot for the equilibrium from conformer **V** to conformer **I**. The K values were 0.97, 0.99, 1.01 at 30 ^{o}C, 50 ^{o}C,

70 ^{o}C as determined by integral ratio of the two conformers. The conformation was assigned with help of titrating 1,4-dinitrobenzene (see ***Supplementary Fig. 12***).

1.2 Guest Binding of Host 1 to Dinitrobenzene 2

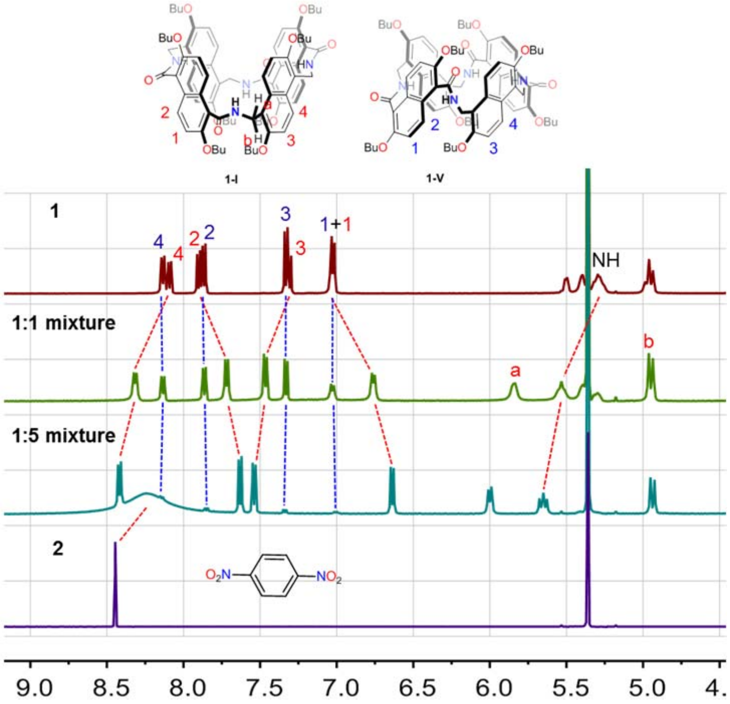

Supplementary Figure 7 Partial ^{1}H NMR spectra (500 MHz, CD_2Cl_2, 2.0 mM, 298 K) of **1**, **2**, and their mixtures in 1:1 and 1:5 ratio.

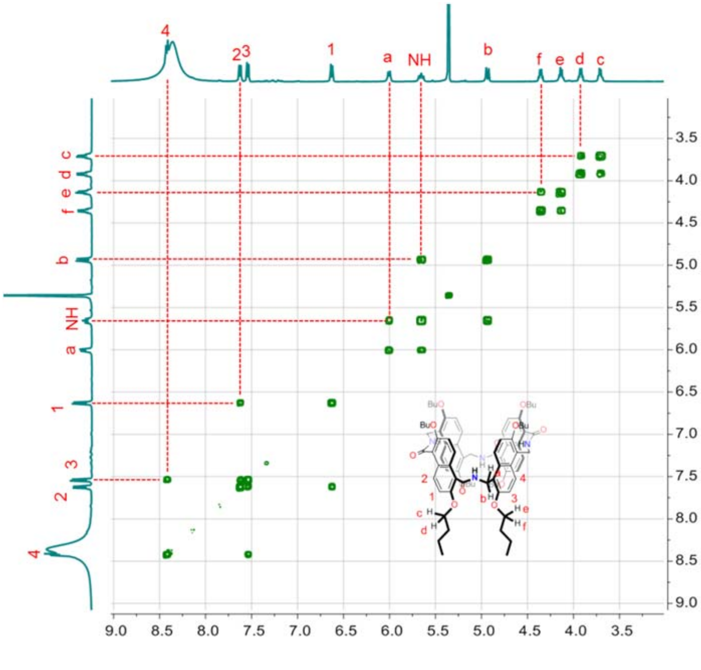

Supplementary Figure 8 Partial ^{1}H-^{1}H COSY spectrum (500 MHz, CD_2Cl_2, 298 K)

of 1:6 mixture in **1** (4 mM) and **2** (24 mM).

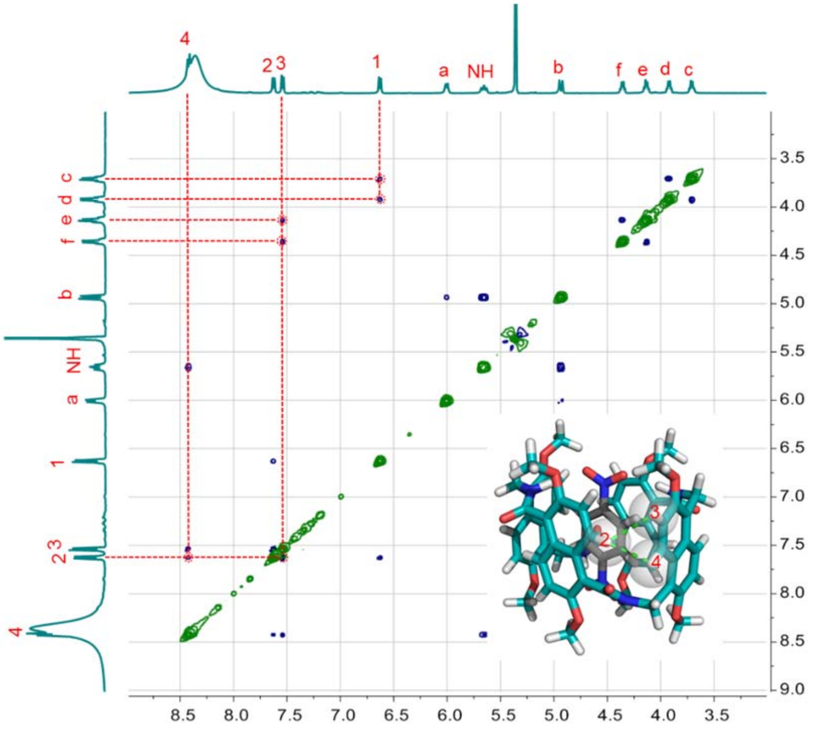

Supplementary Figure 9 Partial ^1H-^1H ROESY spectrum (500 MHz, CD_2Cl_2, 298 K) of 1:6 mixture in **1** (4 mM) and **2** (24 mM). The NOE signal of proton 2 with protons 3 and protons 4 supported conformer **I** was selected by guest **1**.

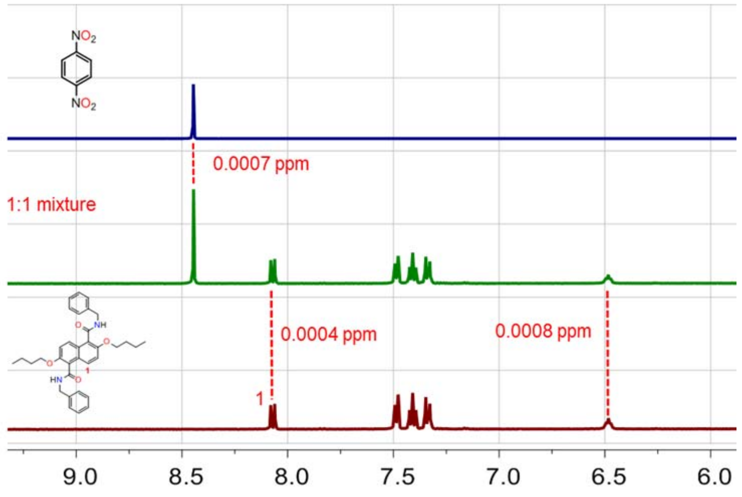

Supplementary Figure 10 Partial ^1H NMR spectra (500 MHz, CD_2Cl_2, 2.0 mM, 298 K) of **2**, an acyclic analogue (**S7**), and their 1:1 mixture. The shift of the ^1H NMR signals of the acyclic analogue when adding guest **2** is very similar to that of conformer **V**, suggesting that there is no specific interactions between conformer **V**

and guest **2**.

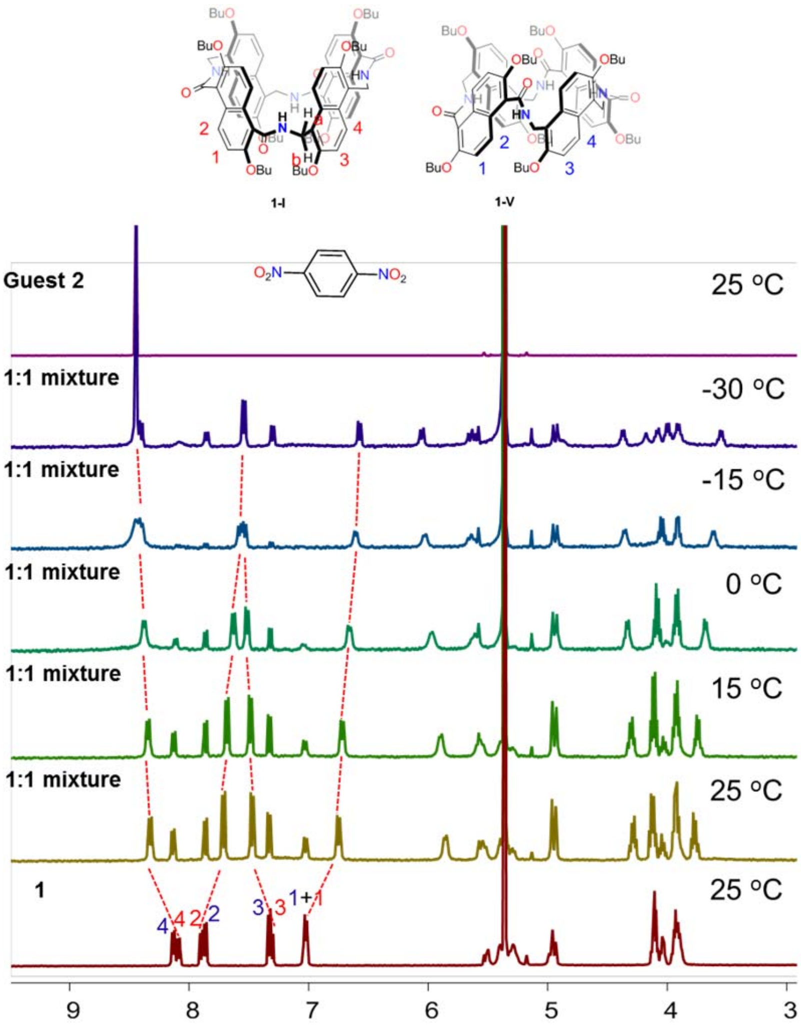

Supplementary Figure 11 Variable-temperature ^{1}H NMR spectra (400 MHz, CD_2Cl_2) of the 1:1 mixture of **1** and guest **2**. As the temperature decreasing, the NMR peak of **1-I** experiences larger chemical shifts**,** this may due to lower solubility of host **1** at lower temperature, and this results in the guest **2** is excess than host **1** in the solution. At -30 °C, the free and bound conformer **I** are cannot be differentiated, suggesting the guest exchange kinetics of complex **2@1-I** is still fast on the NMR timescale even at -30 °C.

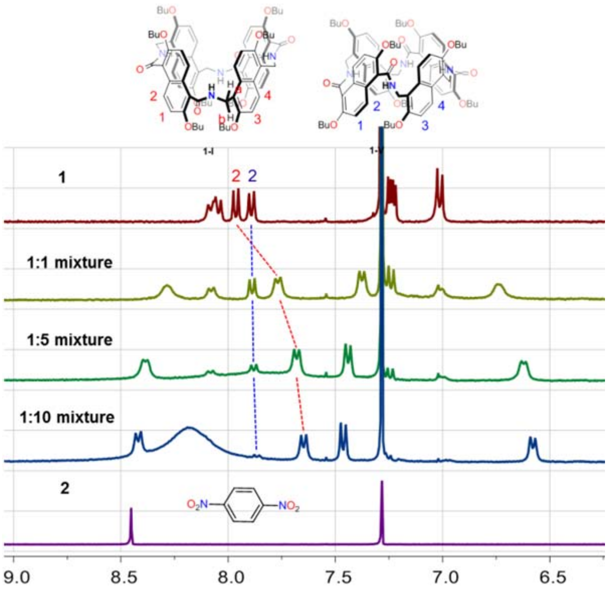

Supplementary Figure 12 Partial ^1H NMR spectra (500 MHz, $CDCl_3$, 2.0 mM, 298 K) of host **1**, guest **2**, and their mixtures in 1:1, 1:5 and 1:10 ratio. Similarly, the conformations were assigned by considering conformer **I** is the predominat binder to **2**.

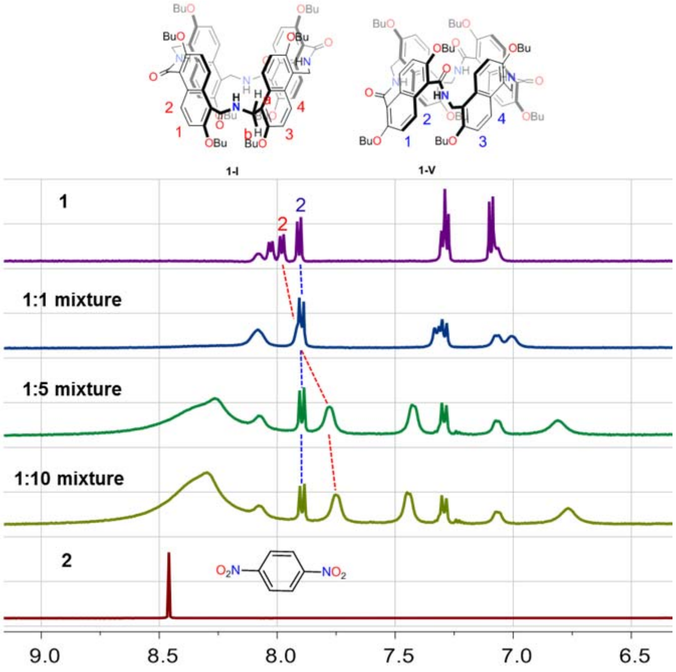

Supplementary Figure 13 Partial ^1H NMR spectra (500 MHz, $Cl_2CDCDCl_2$, 2.0 mM, 298 K) of host **1**, guest **2**, and their mixtures in 1:1, 1:5 and 1:10 ratio. Similarly, the conformations were assigned by considering conformer **I** is the predominat binder

to **2**.

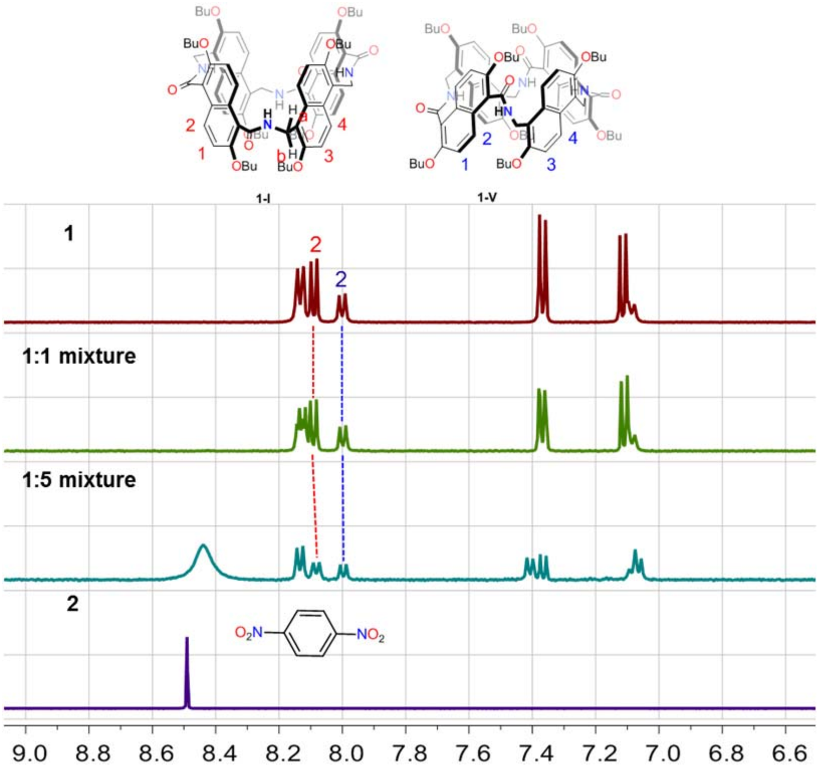

Supplementary Figure 14 Partial ^{1}H NMR spectra (500 MHz, THF-d_8, 1.0 mM, 298 K) of host **1**, guest **2**, and their mixtures in 1:1, and 1:5 ratio. Similarly, the conformations were assigned by considering conformer **I** is the predominat binder to **2**.

1.3 Kinetic Experiments

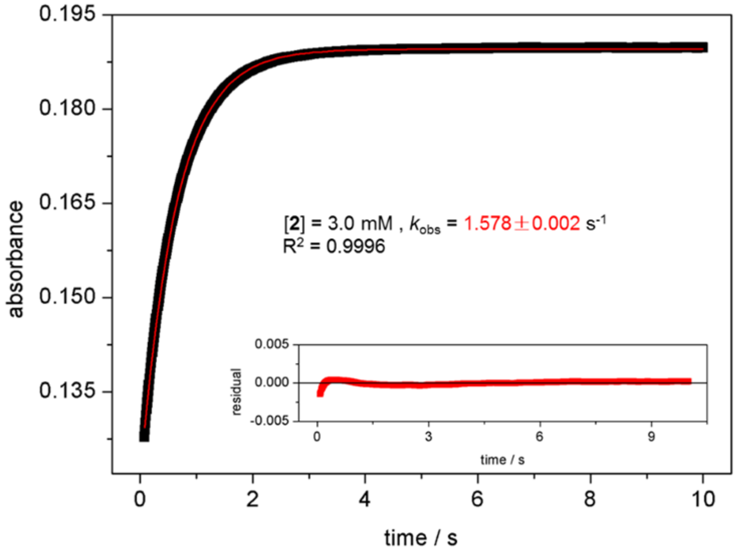

Supplementary Figure 15 Absorbance intensity (480 nm) evolution with time when mixing **1** (2.0 mM in CH_2Cl_2) with **2** (6.0 mM in CH_2Cl_2). The red solid line represents the fitted curve for a single-exponential function (Inset: Residuals for the nonlinear fitted curve).

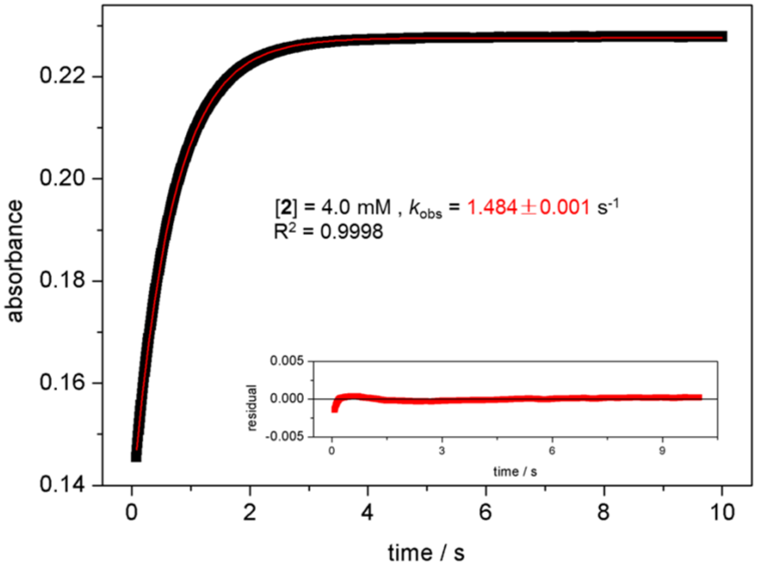

Supplementary Figure 16 Absorbance intensity (480 nm) evolution with time when mixing **1** (2.0 mM in CH_2Cl_2) with **2** (8.0 mM in CH_2Cl_2). The red solid line represents the fitted curve for a single-exponential function (Inset: Residuals for the nonlinear fitted curve).

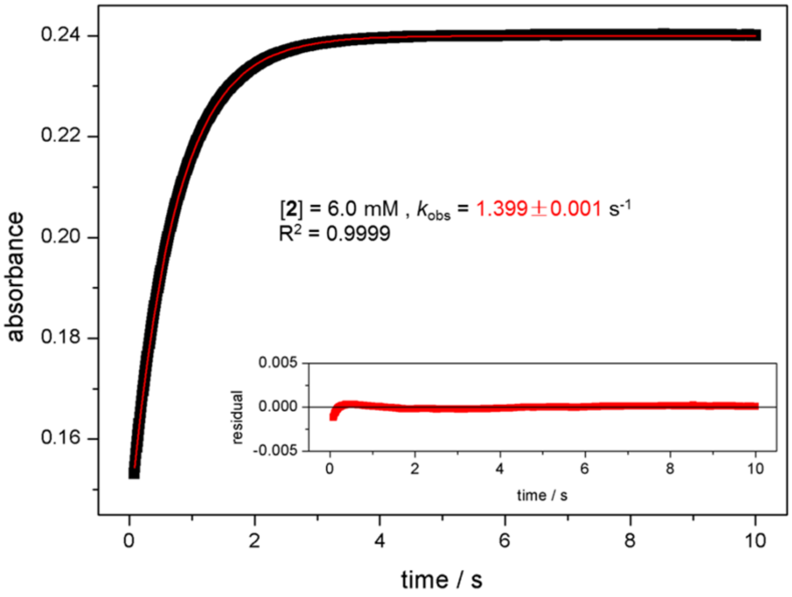

Supplementary Figure 17 Absorbance intensity (480 nm) evolution with time when mixing **1** (2.0 mM in CH_2Cl_2) with **2** (12.0 mM in CH_2Cl_2). The red solid line represents the fitted curve for a single-exponential function (Inset: Residuals for the nonlinear fitted curve).

1.4 Binding Constant Determination

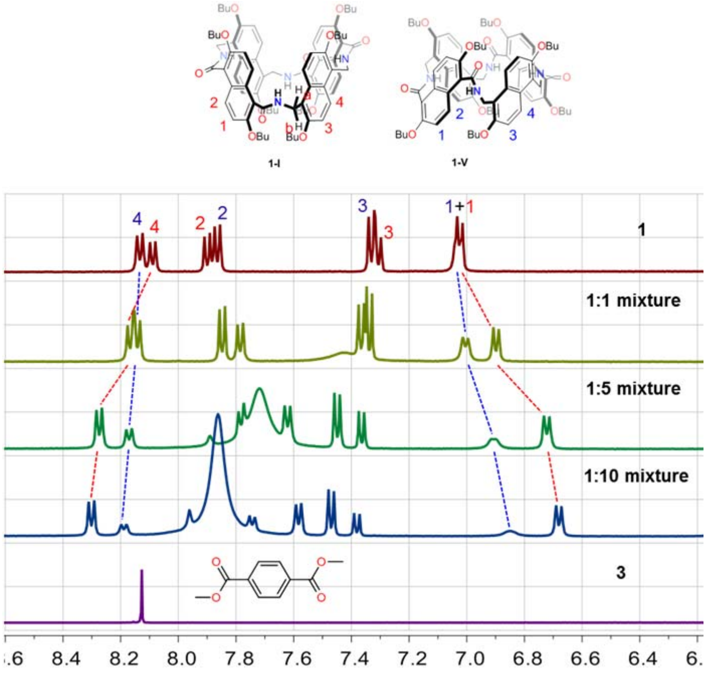

Supplementary Figure 18 Partial ^{1}H NMR spectra (500 MHz, CD_2Cl_2, 2.0 mM, 298 K) of host **1**, guest **3**, and their mixtures in 1:1, 1:5 and 1:10 ratio.

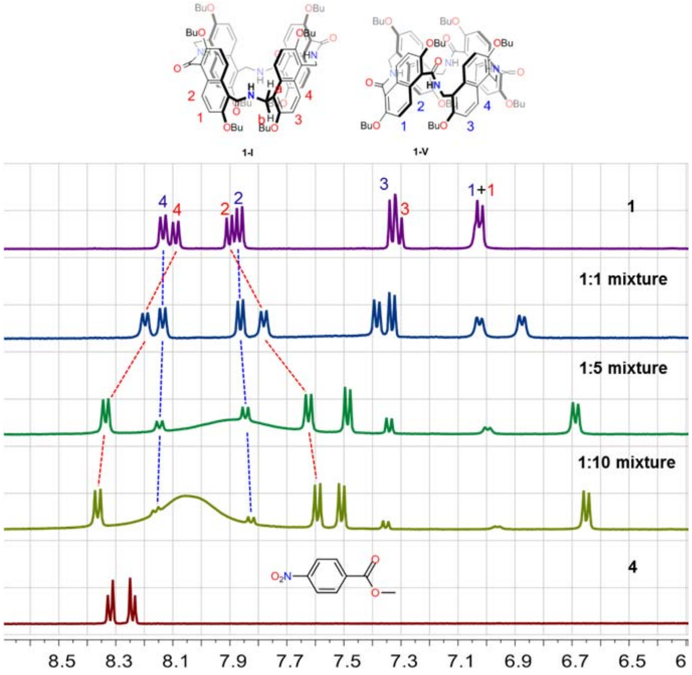

Supplementary Figure 19 Partial ^{1}H NMR spectra (500 MHz, CD_2Cl_2, 2.0 mM, 298 K) of host **1**, guest **4**, and their mixtures in 1:1, 1:5 and 1:10 ratio.

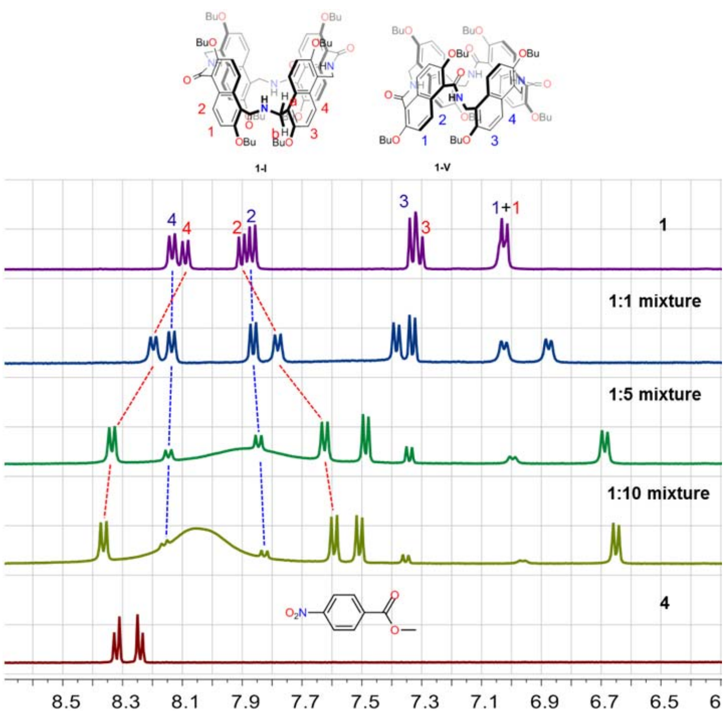

Supplementary Figure 20 Partial ^{1}H NMR spectra (500 MHz, CD_2Cl_2, 2.0 mM, 298 K) of host **1**, guest **5**, and their mixtures in 1:1, 1:5 and 1:10 ratio.

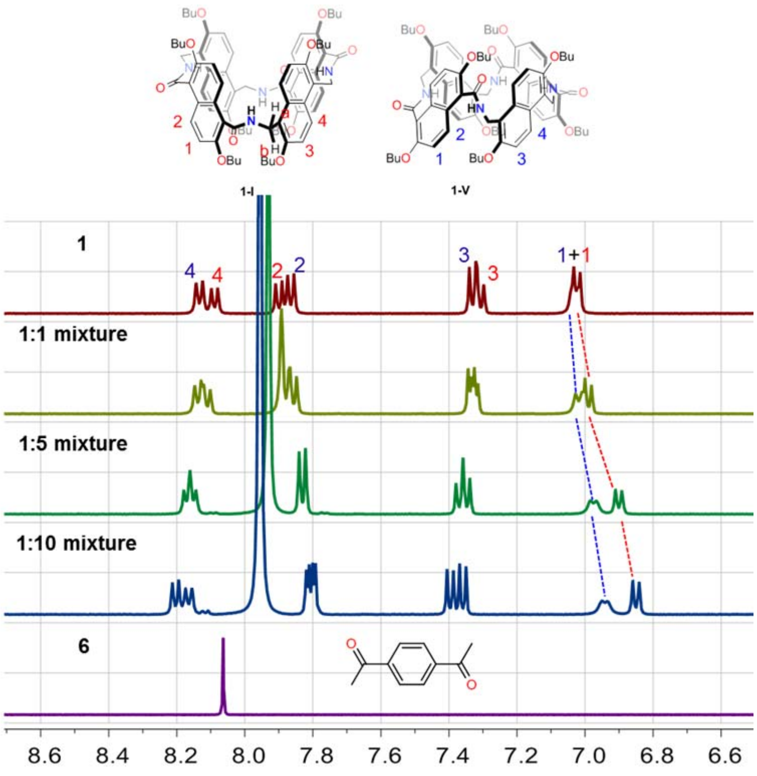

Supplementary Figure 21 Partial ^{1}H NMR spectra (500 MHz, CD_2Cl_2, 2.0 mM, 298 K) of host **1**, guest **6**, and their mixtures in 1:1, 1:5 and 1:10 ratio.

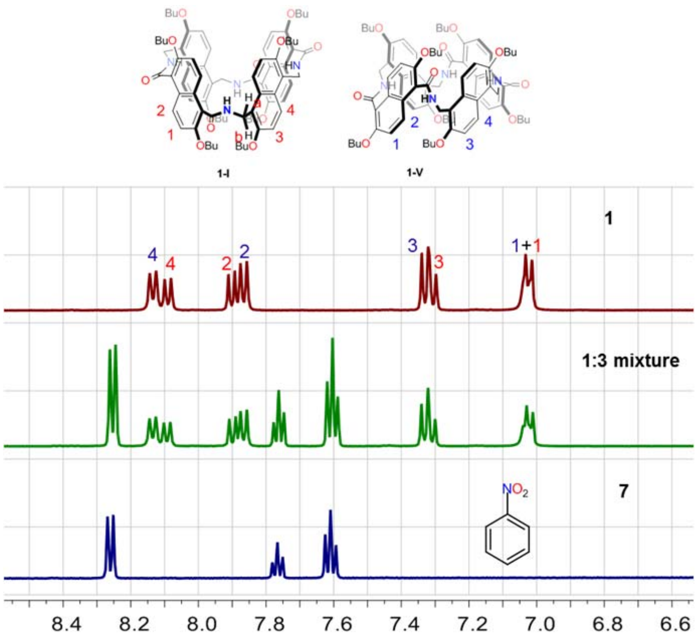

Supplementary Figure 22 Integration of ^{1}H NMR spectra (500 MHz, CD_2Cl_2, 2.0 mM, 298 K) of mixture of host **1** and guest **7** in 1:3 ratio. No obvious shift of both the guest and the guest was detected, suggesting no binding between **1** and **7**.

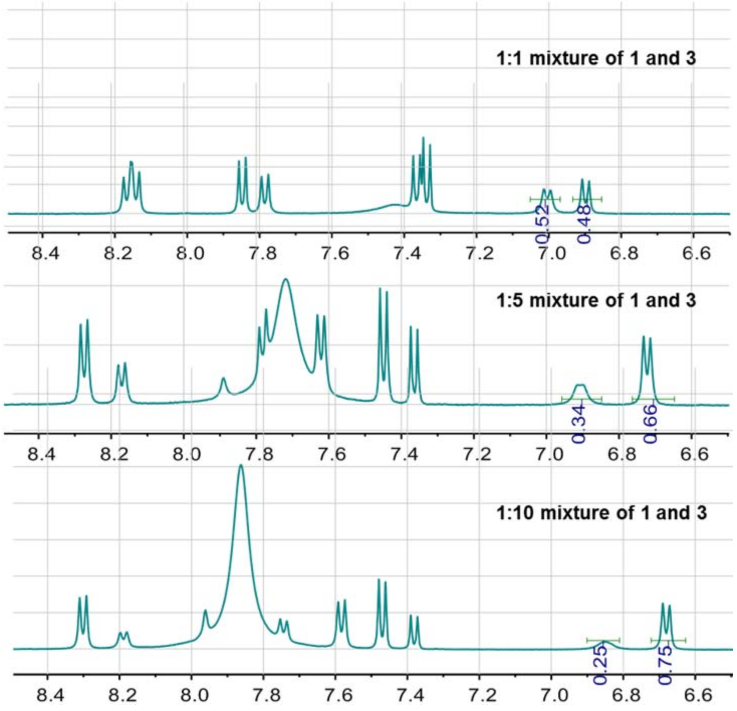

Supplementary Figure 23 Integration of ^{1}H NMR spectra (500 MHz, CD_2Cl_2, 2.0 mM, 298 K) of the mixtures of host **1** and guest **3** in 1:1, 1:5 and 1:10 ratio.

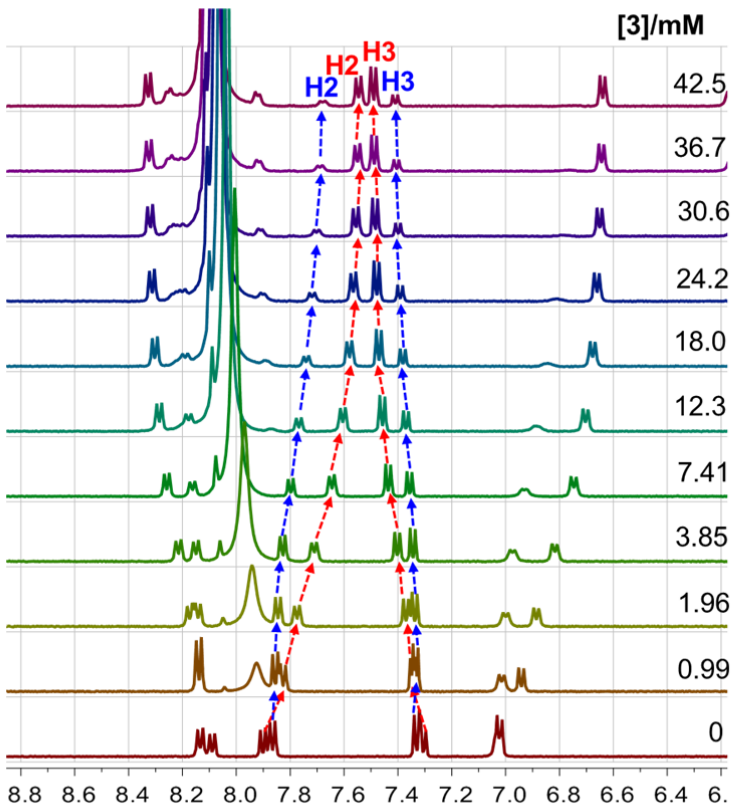

Supplementary Figure 24 Partial ^{1}H NMR spectra (500 MHz, CD_2Cl_2, 25 ^oC) of host **1** (0.5 mM) titrated by guest **3**. From bottom to top, the concentration of **3** was 0~42.5 mM. In order to avoid changing the host concentration when adding large volume of the guest's solution, the same concentration of host **1** (0.5 mM) was also maintained in the stock solution of guests. Thus, the concentration of host **1** in the NMR tube is kept the same during the whole titration process. The same measure was taken for the titration of other guests into the solution of **1**.

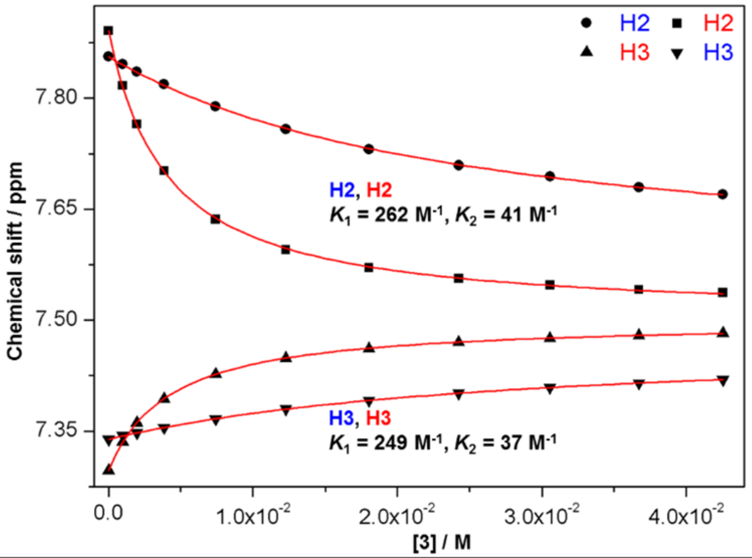

	titration 1			titration 2			average	error
K_1 (M^{-1})	249 (H3)	262 (H2)	256 (average)	251 (H3)	269 (H2)	260 (average)	258	2
K_2 (M^{-1})	37 (H3)	41 (H2)	39 (average)	38 (H3)	43 (H2)	41 (average)	40	1
R^2	0.9999	0.9999		0.9998	0.9999			

Supplementary Figure 25 Non-linear curve-fitting for the NMR data between guest **3** and host **1** in CD_2Cl_2 at 298 K according to equations (20) and (21) in **Supplementary Note 1** (equations (6) and (7) in the maintext) by using a global fitting method with sharing parameters.

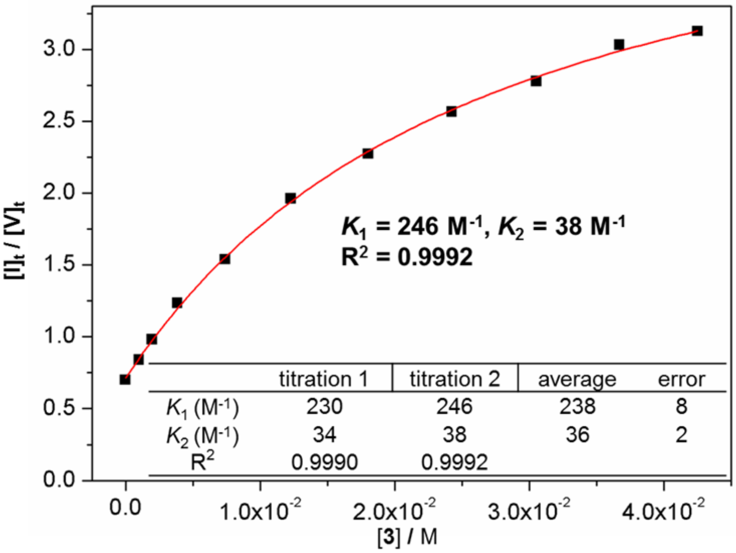

	titration 1	titration 2	average	error
K_1 (M^{-1})	230	246	238	8
K_2 (M^{-1})	34	38	36	2
R^2	0.9990	0.9992		

Supplementary Figure 26 Non-linear curve-fitting for the NMR data between **3** and host **1** in CD_2Cl_2 at 298 K according to equation (25) in **Supplementary Note 1** (equation (8) in the maintext).

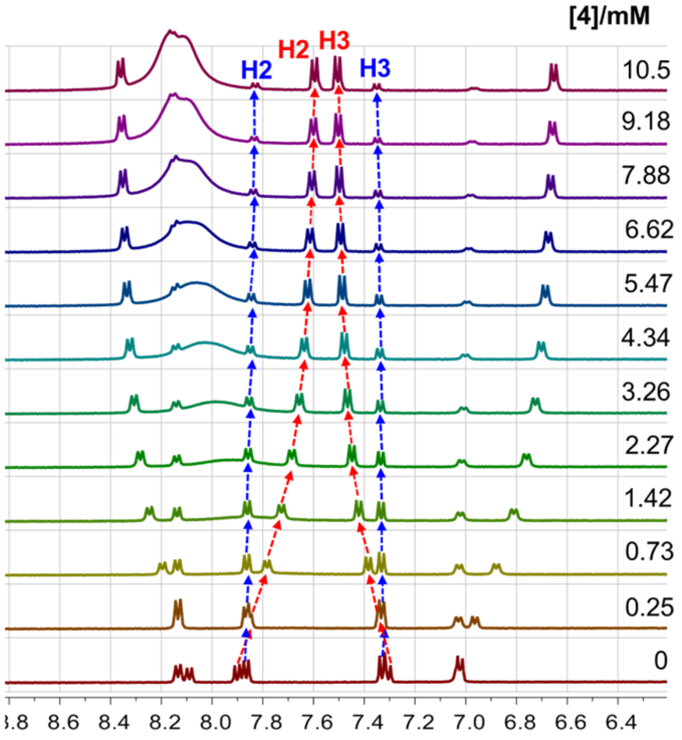

Supplementary Figure 27 Partial ^{1}H NMR spectra (500 MHz, CD_2Cl_2, 25 ^oC) of host **1** (0.5 mM) titrated by guest **4** . From bottom to top, the concentration of **4** was 0~10.5 mM.

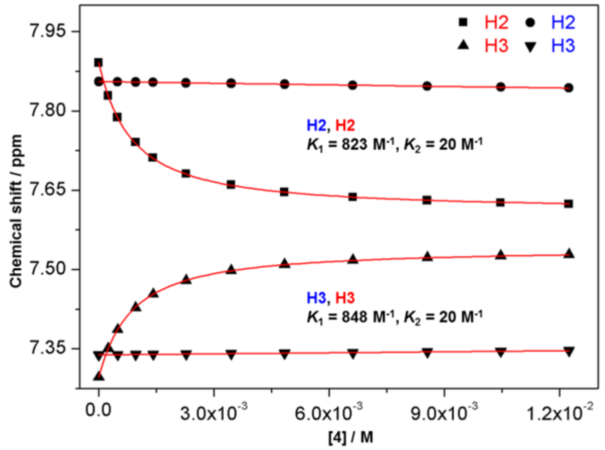

	titration 1			titration 2			average	error
K_1 (M^{-1})	848 (H3)	823 (H2)	836 (average)	848 (H3)	860 (H2)	854 (average)	845	9
K_2 (M^{-1})	20 (H3)	20 (H2)	20 (average)	13 (H3)	25 (H2)	19 (average)	20	1
R^2	0.9999	0.9999		0.9999	0.9999			

Supplementary Figure 28 Non-linear curve-fitting for the NMR data between guest **4** and host **1** in CD_2Cl_2 at 298 K according to equations (20) and (21) in

Supplementary Note 1 (equations (6) and (7) in the maintext) by using a global fitting method with sharing parameters.

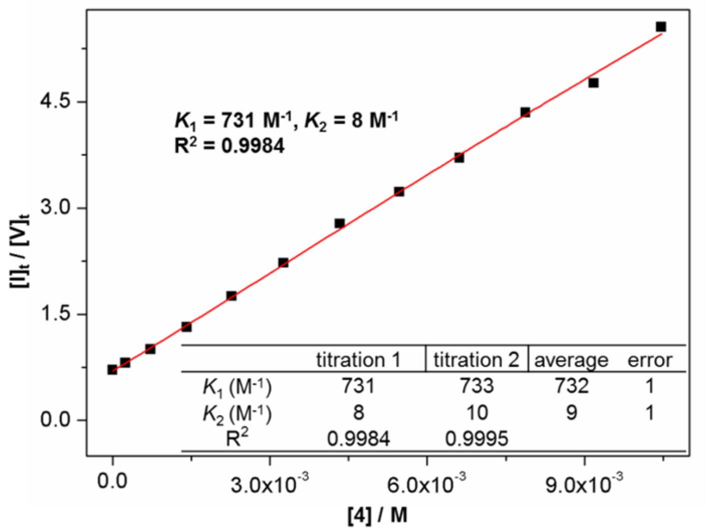

Supplementary Figure 29 Non-linear curve-fitting for the NMR data between **4** and host **1** in CD_2Cl_2 at 298 K according to equation (25) in **Supplementary Note 1** (equation (8) in the maintext).

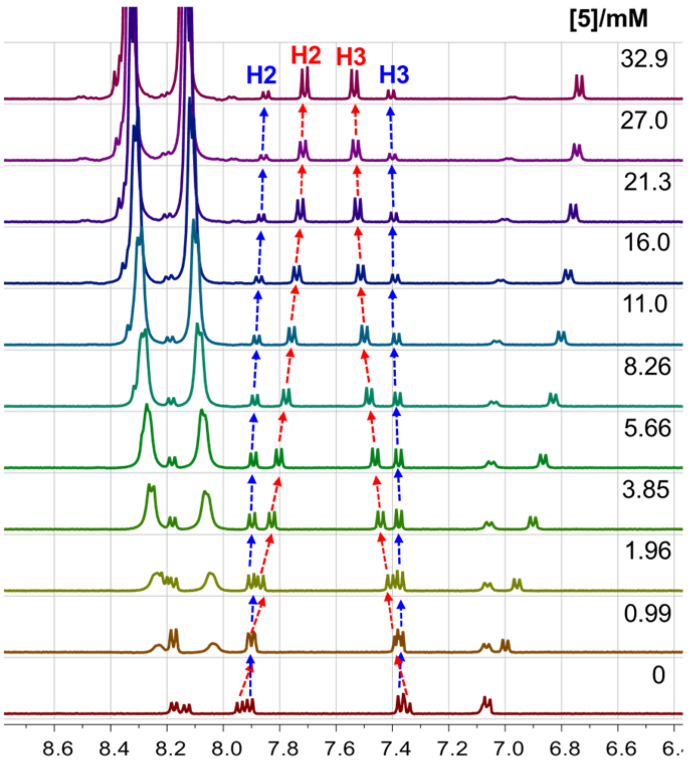

Supplementary Figure 30 Partial ^{1}H NMR spectra (500 MHz, CD_2Cl_2, 25 °C) of host **1** (0.5 mM) titrated by guest **5**. From bottom to top, the concentration of **5** was 0~32.9 mM.

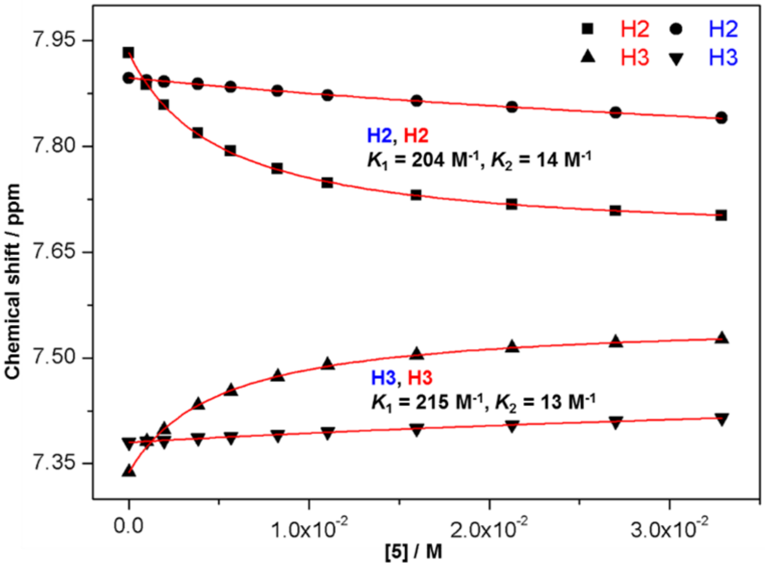

	titration 1			titration 2			average	error
K_1 (M^{-1})	215 (H3)	204 (H2)	210 (average)	178 (H3)	173 (H2)	176 (average)	193	17
K_2 (M^{-1})	13 (H3)	14 (H2)	14 (average)	10 (H3)	10 (H2)	10 (average)	12	2
R^2	0.9999	0.9999		0.9999	0.9999			

Supplementary Figure 31 Non-linear curve-fitting for the NMR data between guest **5** and host **1** in CD_2Cl_2 at 298 K according to equations (20) and (21) in **Supplementary Note 1** (equations (6) and (7) in the maintext) by using a global fitting method with sharing parameters.

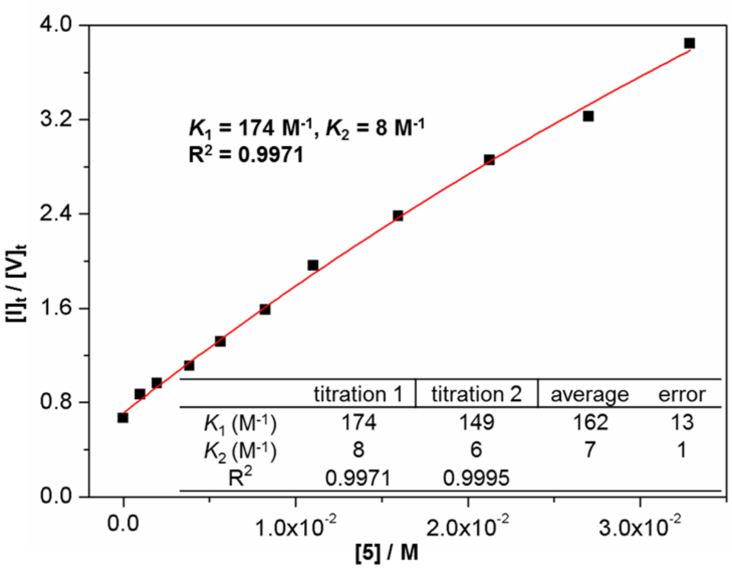

Supplementary Figure 32 Non-linear curve-fitting for the NMR data between **5** and host **1** in CD_2Cl_2 at 298 K according to equation (25) in **Supplementary Note 1** (equation (8) in the maintext).

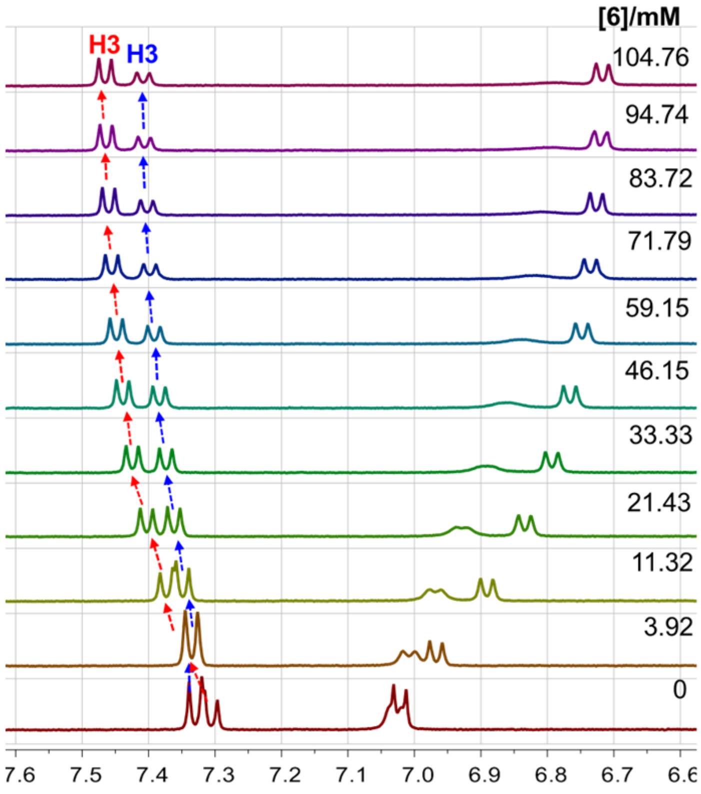

Supplementary Figure 33 Partial ^{1}H NMR spectra (500 MHz, CD_2Cl_2, 25 ^oC) of host **1** (0.5 mM) titrated by guest **6**. From bottom to top, the concentration of **6** was 0~104.76 mM.

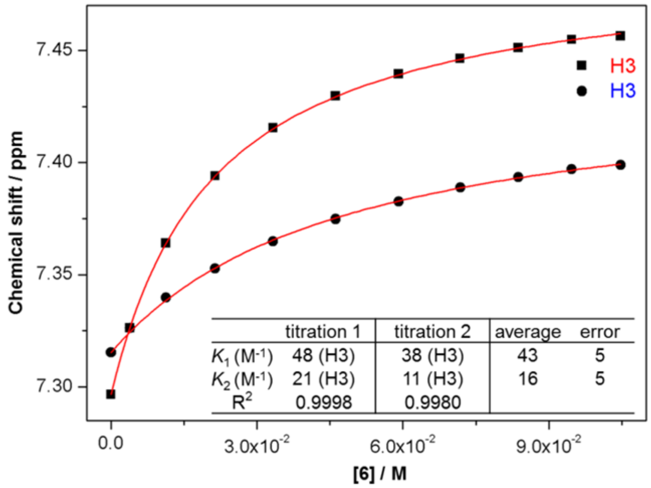

Supplementary Figure 34 Non-linear curve-fitting for the NMR data between guest **6** and host **1** in CD_2Cl_2 at 298 K according to equations (20) and (21) in **Supplementary Note 1** (equations (6) and (7) in the maintext) by using a global fitting method with sharing parameters.

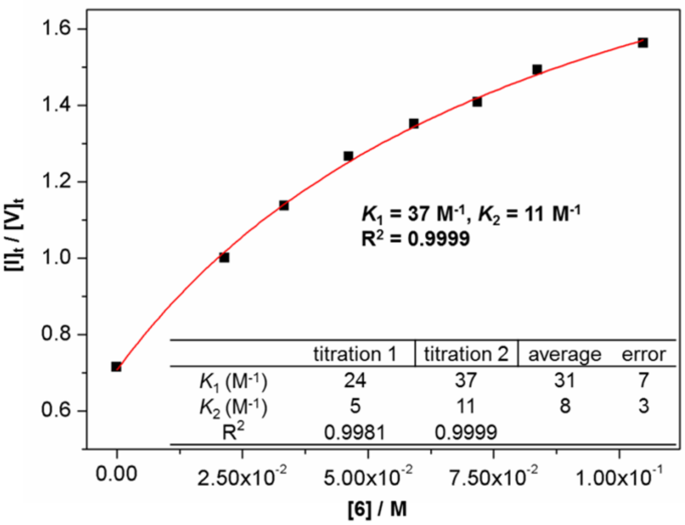

Supplementary Figure 35 Non-linear curve-fitting for the NMR data between **6** and host **1** in CD_2Cl_2 at 298 K according to equation (25) in **Supplementary Note 1** (equation (8) in the maintext).

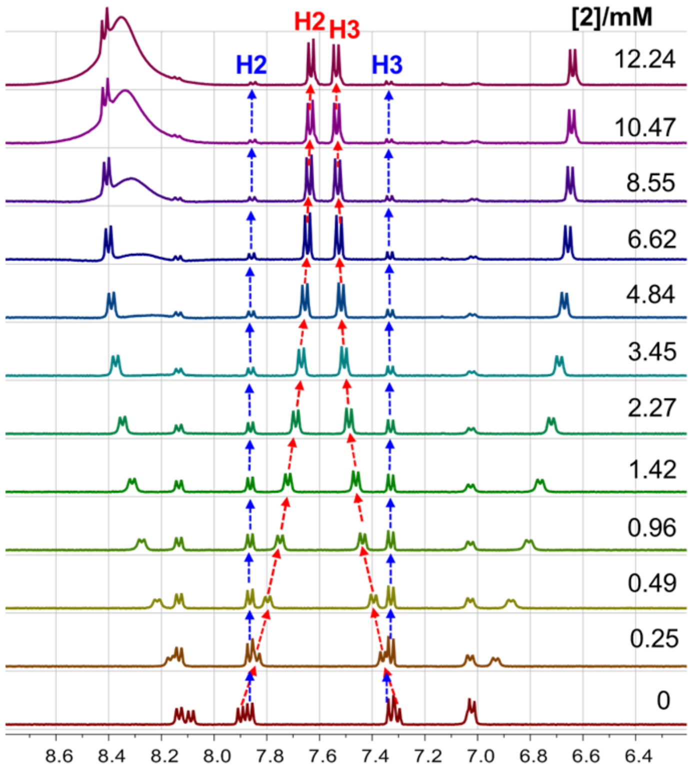

Supplementary Figure 36 Partial ^{1}H NMR spectra (500 MHz, CD_2Cl_2, 25 ^{o}C) of host **1** (0.5 mM) titrated by guest **2**. From bottom to top, the concentration of **2** was 0~12.24 mM. Aromatic protons of **H2** and **H3** were monitored during the titration for the calculation of binding constants.

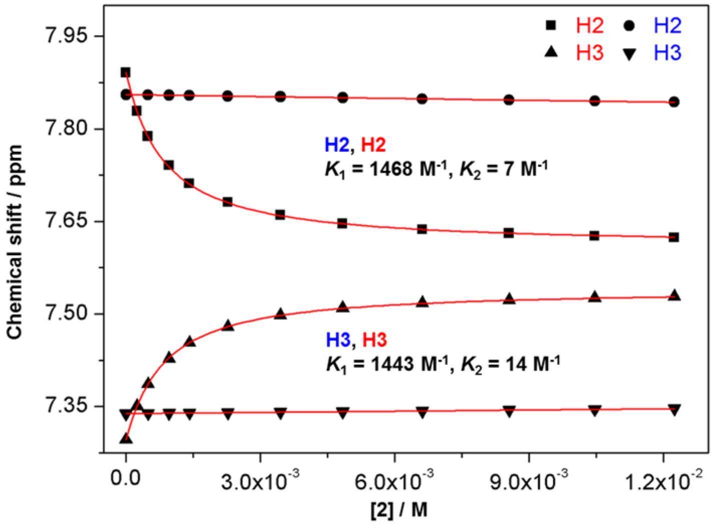

	titration 1			titration 2			average	error
K_1 (M^{-1})	1443 (H3)	1468 (H2)	1456 (average)	1510 (H3)	1470 (H2)	1490 (average)	1473	17
K_2 (M^{-1})	14 (H3)	7 (H2)	10 (average)	0 (H3)	16 (H2)	8 (average)	9	1
R^2	0.9999	0.9999		0.9999	0.9999			

Supplementary Figure 37 Non-linear curve-fitting for the NMR data between guest **2** and host **1** in CD_2Cl_2 at 298 K according to equations (20) and (21) in **Supplementary Note 1** (equations (6) and (7) in the maintext) by using a global fitting method with sharing parameters.

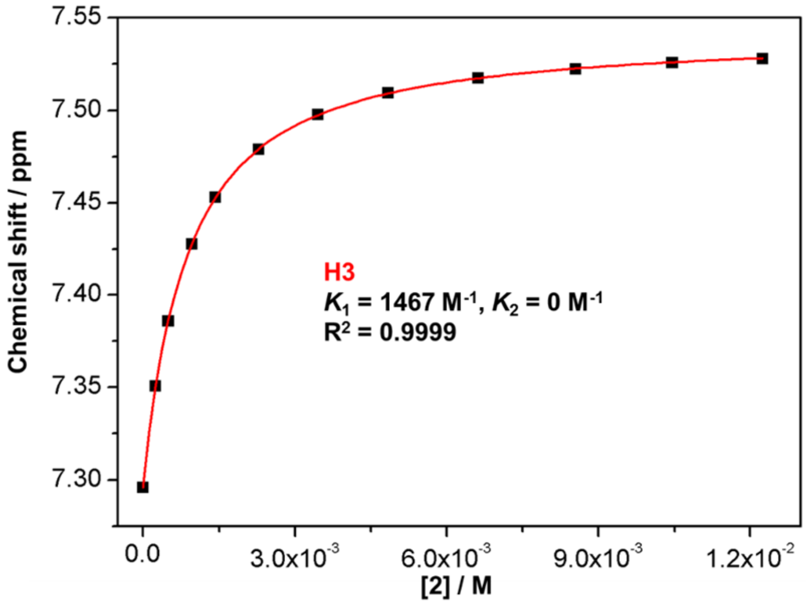

Supplementary Figure 38 Non-linear curve-fitting for the NMR data between guest **2** and host **1** in CD_2Cl_2 at 298 K according to equation (20) in **Supplementary Note 1** by setting $K_2 = 0$.

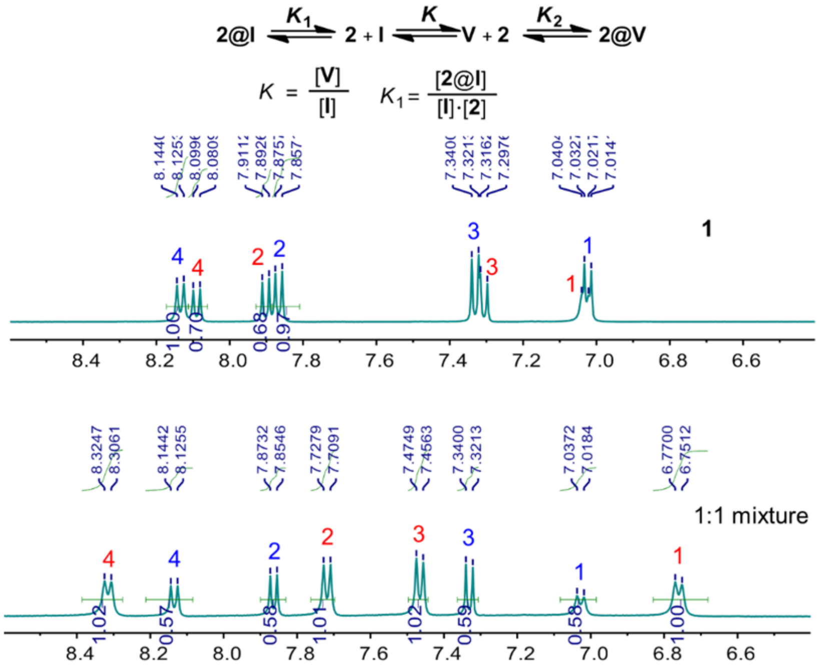

Supplementary Figure 39 Top: complexation equilibria and the expression of the two equilibria between two conformers and the complexation between **2** and conformer **I**. Bottom: partial ^{1}H NMR spectra (500 MHz, CD_2Cl_2, 2.0 mM, 298 K) of free host **1** and 1:1 mixture of guest **2** and host **1**. As K_1 is much higher than K_2, we can assume $K_2 = 0$. Then, K_1 can be estimated by the integration method.

The reciprocal of equilibrium constant K is calculated as followed by using the integrals of protons H2 for free host:

$1/K = [(0.68 / 1.65) \times 2.0 \times 10^{-3}] / [(0.97 / 1.65) \times 2.0 \times 10^{-3}] = 0.7.$

In the 1:1 mixture of **2** and **1,** the following calculation can be performed by using the integrals of H_1 of complexed (conformer **I**) and uncomplexed (conformer **V**) **1**:

[V] $= (0.59 / 1.61)*2.0 \times 10^{-3}\ M^{-1} = 0.733 \times 10^{-3}\ M^{-1}$

[I] = **[V]** $\times 1 / K = 0.734 \times 10^{-3}\ M^{-1} \times 0.7 = 0.513 \times 10^{-3}\ M^{-1}$

[2@I] = **[1] - [I] - [V]** $= (2.000 - 0.733 - 0.513) = 0.754 \times 10^{-3}\ M^{-1}$

[2] $= (2.000 - 0.754) \times 10^{-3}\ M^{-1} = 1.246 \times 10^{-3}\ M^{-1}$

$K_1 = 0.754 \times 10^{-3} / (0.513 \times 10^{-3} \times 1.246 \times 10^{-3})\ M^{-1} = 1180\ M^{-1}$

Similarly, from the integrals of protons H_2, H_3 and H_4 of complexed and uncomplexed host **1**, K_1 was calculated to be 1276 M^{-1}, 1197 M^{-1} and 1172 M^{-1}, respectively.

Finally, $K_1 = (1180 + 1276 + 1197 + 1172) / 4 = 1206 (\pm 41)\ M^{-1}$.

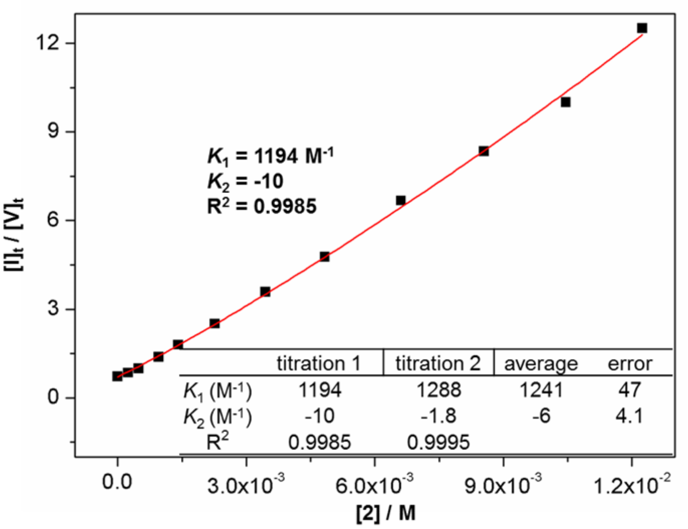

Supplementary Figure 40 Non-linear curve-fitting for the NMR data (***Supplementary Fig. 36***) between **2** and host **1** in CD_2Cl_2 at 298 K according to equation (25) in **Supplementary Note 1** (equation (8) in maintext).

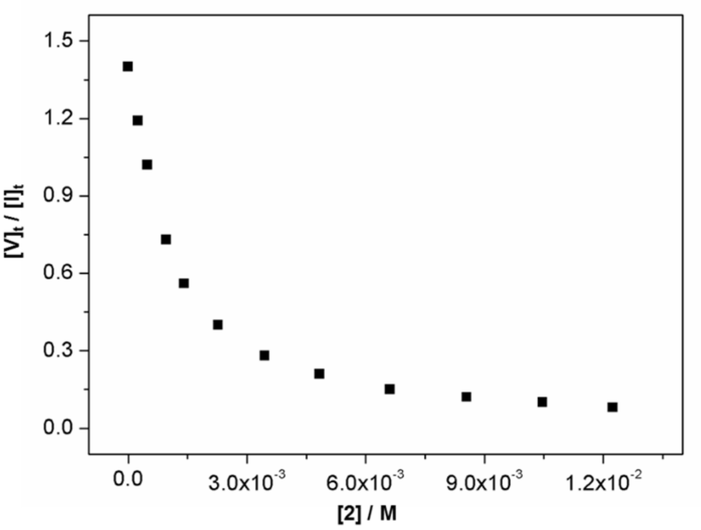

Supplementary Figure 41 The scatter plot of $[\mathbf{V}]_t / [\mathbf{I}]_t$ with [**2**]. Note: In **Supplementary Figure 40**, it seems to be linear relationship between $[\mathbf{I}]_t / [\mathbf{V}]_t$ and [**2**], and one may think that the titration did not reach saturation. However, the obvious inflection in scatter plot of $[\mathbf{V}]_t / [\mathbf{I}]_t$ with the function of [**2**] (right figure) indicates the titration is already saturated.

2. Supplementary Note 1

Derivation of Fitting Equations of Competitive Complexation.

$$\mathbf{G@I} \underset{}{\overset{K_1}{\leftleftharpoons}} \mathbf{G + I} \overset{K}{\rightleftharpoons} \mathbf{V + G} \overset{K_2}{\rightleftharpoons} \mathbf{G@V}$$

For the reaction scheme shown above, the association constants (K_1 and K_2) for these equilibria are defined as followed:

$$K_1 = \frac{[\boldsymbol{G@I}]}{[\boldsymbol{I}][\boldsymbol{G}]} \quad (1)$$

$$K_2 = \frac{[\boldsymbol{G@V}]}{[\boldsymbol{V}][\boldsymbol{G}]} \quad (2)$$

Thus,

$$[\boldsymbol{G@I}] = K_1[\boldsymbol{I}][\boldsymbol{G}] \quad (3)$$

$$[\boldsymbol{G@V}] = K_2[\boldsymbol{V}][\boldsymbol{G}] \quad (4)$$

It can be assumed that the equilibrium between **I** and **V** is not affected by the addition of a guest, and the conformational equilibrium constant is kept constant:

$$K = \frac{[\boldsymbol{V}]}{[\boldsymbol{I}]} \quad (5)$$

The total molar concentration of each conformer $[\boldsymbol{I}]_t$ and $[\boldsymbol{V}]_t$ are the sum of free **I** = $[\boldsymbol{I}]$ and bound **I** = $[\boldsymbol{G@I}]$, free **V** = $[\boldsymbol{V}]$ and bound **V** = $[\boldsymbol{G@V}]$, respectively.

$$[\boldsymbol{I}]_t=[\boldsymbol{I}]+[\boldsymbol{G@I}]=[\boldsymbol{I}]+K_1[\boldsymbol{I}][\boldsymbol{G}] \quad (6)$$

$$[\boldsymbol{V}]_t=[\boldsymbol{V}]+[\boldsymbol{G@V}]=[\boldsymbol{V}]+K_2[\boldsymbol{V}][\boldsymbol{G}] \quad (7)$$

By introducing equilibrium constant K as follows, the total concentration of the host $[\boldsymbol{H}]_t$ and the guest $[\boldsymbol{G}]_t$ can be formulated as (8) and (9):

$$[\boldsymbol{H}]_t=[\boldsymbol{I}]_t+[\boldsymbol{V}]_t=[\boldsymbol{I}]+K_1[\boldsymbol{I}][\boldsymbol{G}]+[\boldsymbol{V}]+K_2[\boldsymbol{V}][\boldsymbol{G}]=[\boldsymbol{I}]+K_1[\boldsymbol{I}][\boldsymbol{G}]+K[\boldsymbol{I}]+KK_2[\boldsymbol{I}][\boldsymbol{G}] \quad (8)$$

$$[\boldsymbol{G}]_t=[\boldsymbol{G}]+[\boldsymbol{G}@\boldsymbol{I}]+[\boldsymbol{G}@\boldsymbol{V}]=[\boldsymbol{G}]+K_1[\boldsymbol{I}][\boldsymbol{G}]+KK_2[\boldsymbol{I}][\boldsymbol{G}]=[\boldsymbol{G}](1+K_1[\boldsymbol{I}]+KK_2[\boldsymbol{I}])=[\boldsymbol{G}][1+[\boldsymbol{I}](K_1+KK_2)] \quad (9)$$

Combining like terms from (8) gives us (10):

$$[\boldsymbol{H}]_t=[\boldsymbol{I}](1+K+K_1[\boldsymbol{G}]+KK_2[\boldsymbol{G}]) \ (10)$$

Isolating $[\boldsymbol{I}]$ from (10) gives us (11):

$$[\boldsymbol{I}]=\frac{[\boldsymbol{H}]_t}{1+K+K_1[\boldsymbol{G}]+KK_2[\boldsymbol{G}]} \quad (11)$$

Inserting (11) into (9) gives us (12):

$$[\boldsymbol{G}]_t=[\boldsymbol{G}]\left(1+\frac{[\boldsymbol{H}]_t(K_1+KK_2)}{1+K+K_1[\boldsymbol{G}]+KK_2[\boldsymbol{G}]}\right)=[\mathbf{G}]\left(\frac{1+K+K_1[\boldsymbol{G}]+KK_2[\boldsymbol{G}]+[\boldsymbol{H}]_t(K_1+KK_2)}{1+K+K_1[\boldsymbol{G}]+KK_2[\boldsymbol{G}]}\right) \quad (12)$$

Rearranging (12) gives us (13):

$$[\boldsymbol{G}]\big(1+K+K_1[\boldsymbol{G}]+KK_2[\boldsymbol{G}]+[\boldsymbol{H}]_t(K_1+KK_2)\big)-[\boldsymbol{G}]_t(1+K+K_1[\boldsymbol{G}]+KK_2[\boldsymbol{G}])=0 \quad (13)$$

Expanding (13) gives us (14):

$$[\boldsymbol{G}]+K[\boldsymbol{G}]+K_1[\boldsymbol{G}]^2+KK_2[\boldsymbol{G}]^2+[\boldsymbol{H}]_t(K_1+KK_2)[\boldsymbol{G}]-[\boldsymbol{G}]_t(1+K)-[\boldsymbol{G}]_t(K_1+KK_2)[\boldsymbol{G}]=0 \ (14)$$

Combining like terms from (14) gives us the quadratic (15):

$$(K_1+KK_2)[\boldsymbol{G}]^2+\big(1+K+(K_1+KK_2)([\boldsymbol{H}]_t-[\boldsymbol{G}]_t)\big)[\boldsymbol{G}]-[\boldsymbol{G}]_t(1+K)=0 \quad (15)$$

Solving equations (15) gives us (16).

$$[\boldsymbol{G}]=\frac{-\left(1+K+(K_1+KK_2)([\boldsymbol{H}]_\mathrm{t}-[\boldsymbol{G}]_\mathrm{t})\right)+\sqrt{\left(1+K+(K_1+KK_2)([\boldsymbol{H}]_\mathrm{t}-[\boldsymbol{G}]_\mathrm{t})\right)^2+4(K_1+KK_2)(1+K)[\boldsymbol{G}]_\mathrm{t}}}{2(K_1+KK_2)} \qquad (16)$$

In fast exchanging systems, the observed chemical shifts (δ_obs) of the proton of conformer **I** becomes the weighted average of the free (δ_F) and bound (δ_B) states[2], and then (17) can be derived.

$$\delta_\mathrm{obs}=x_\mathrm{F}\delta_\mathrm{F}+x_\mathrm{B}\delta_\mathrm{B}=\delta_\mathrm{F}\frac{[\boldsymbol{I}]}{[\boldsymbol{I}]+[\boldsymbol{G@I}]}+\delta_\mathrm{B}\frac{[\boldsymbol{G@I}]}{[\boldsymbol{I}]+[\boldsymbol{G@I}]}=\delta_\mathrm{F}\frac{[\boldsymbol{I}]}{[\boldsymbol{I}]+K_1[\boldsymbol{I}][\boldsymbol{G}]}+\delta_\mathrm{B}\frac{K_1[\boldsymbol{I}][\boldsymbol{G}]}{[\boldsymbol{I}]+K_1[\boldsymbol{I}][\boldsymbol{G}]}=\frac{\delta_\mathrm{F}+\delta_\mathrm{B}K_1[\boldsymbol{G}]}{1+K_1[\boldsymbol{G}]} \qquad (17)$$

where x_F and x_B are the mole fraction of the free component and the complex, respectively.

By combining (16) with (17) to get a theoretical expression (18) of δ_obs of conformer **I** as the function of $[\boldsymbol{G}]_\mathrm{t}$:

$$\delta_\mathrm{obs}=\frac{\delta_\mathrm{F}+\delta_\mathrm{B}K_1\dfrac{-\left(1+K+(K_1+KK_2)([\boldsymbol{H}]_\mathrm{t}-[\boldsymbol{G}]_\mathrm{t})\right)+\sqrt{\left(1+K+(K_1+KK_2)([\boldsymbol{H}]_t-[\boldsymbol{G}]_\mathrm{t})\right)^2+4(K_1+KK_2)(1+K)[\boldsymbol{G}]_\mathrm{t}}}{2(K_1+KK_2)}}{1+K_1\dfrac{-\left(1+K+(K_1+KK_2)([\boldsymbol{H}]_\mathrm{t}-[\boldsymbol{G}]_\mathrm{t})\right)+\sqrt{\left(1+K+(K_1+KK_2)([\boldsymbol{H}]_\mathrm{t}-[\boldsymbol{G}]_\mathrm{t})\right)^2+4(K_1+KK_2)(1+K)[\boldsymbol{G}]_\mathrm{t}}}{2(K_1+KK_2)}} \qquad (18)$$

Modifying (18) gives us (19):

$$\delta_\mathrm{obs}=\frac{\dfrac{2(K_1+KK_2)\delta_\mathrm{F}+\delta_\mathrm{B}K_1\left\{-\left(1+K+(K_1+KK_2)([\boldsymbol{H}]_\mathrm{t}-[\boldsymbol{G}]_\mathrm{t})\right)+\sqrt{\left(1+K+(K_1+KK_2)([\boldsymbol{H}]_\mathrm{t}-[\boldsymbol{G}]_\mathrm{t})\right)^2+4(K_1+KK_2)(1+K)[\boldsymbol{G}]_\mathrm{t}}\right\}}{2(K_1+KK_2)}}{\dfrac{2(K_1+KK_2)+K_1\left\{-\left(1+K+(K_1+KK_2)([\boldsymbol{H}]_\mathrm{t}-[\boldsymbol{G}]_\mathrm{t})\right)+\sqrt{\left(1+K+(K_1+KK_2)([\boldsymbol{H}]_\mathrm{t}-[\boldsymbol{G}]_\mathrm{t})\right)^2+4(K_1+KK_2)(1+K)[\boldsymbol{G}]_\mathrm{t}}\right\}}{2(K_1+KK_2)}} \qquad (19)$$

Then the final theoretical expression of δ_{obs} of conformer **I** with $[\boldsymbol{G}]_t$ (20) can be get by reducing fraction of (19):

$$\delta_{\text{obs}}=\frac{2(K_1+KK_2)\delta_{\text{F}}+\delta_{\text{B}}K_1\left\{-\left(1+K+(K_1+KK_2)([\boldsymbol{H}]_{\text{t}}-[\boldsymbol{G}]_{\text{t}})\right)+\sqrt{\left(1+K+(K_1+KK_2)([\boldsymbol{H}]_{\text{t}}-[\boldsymbol{G}]_{\text{t}})\right)^2+4(K_1+KK_2)(1+K)[\boldsymbol{G}]_{\text{t}}}\right\}}{2(K_1+KK_2)+K_1\left\{-\left(1+K+(K_1+KK_2)([\boldsymbol{H}]_{\text{t}}-[\boldsymbol{G}]_{\text{t}})\right)+\sqrt{\left(1+K+(K_1+KK_2)([\boldsymbol{H}]_{\text{t}}-[\boldsymbol{G}]_{\text{t}})\right)^2+4(K_1+KK_2)(1+K)[\boldsymbol{G}]_{\text{t}}}\right\}} \quad (20)$$

Similarly, a theoretical expression of δ_{obs} of conformer **V** with $[\boldsymbol{G}]_{\text{t}}$ (21) can be easily derived.

$$\delta_{\text{obs}}=\frac{2(K_1/K+K_2)\delta_{\text{F}}+\delta_{\text{B}}K_2\left\{-\left(1+1/K+(K_1/K+K_2)([\boldsymbol{H}]_{\text{t}}-[\boldsymbol{G}]_{\text{t}})\right)+\sqrt{\left(1+1/K+(K_1/K+K_2)([\boldsymbol{H}]_{\text{t}}-[\boldsymbol{G}]_{\text{t}})\right)^2+4(K_1/K+K_2)(1+1/K)[\boldsymbol{G}]_{\text{t}}}\right\}}{2(K_1/K+K_2)+K_2\left\{-\left(1+1/K+(K_1/K+K_2)([\boldsymbol{H}]_{\text{t}}-[\boldsymbol{G}]_{\text{t}})\right)+\sqrt{\left(1+1/K+(K_1/K+K_2)([\boldsymbol{H}]_{\text{t}}-[\boldsymbol{G}]_{\text{t}})\right)^2+4(K_1/K+K_2)(1+1/K)[\boldsymbol{G}]_{\text{t}}}\right\}} \quad (21)$$

Nonlinear curve-fitting method through the above equations was then used to obtain the association constants (K_1, K_2).

The conformational interconversion between two conformers of **1** is slow on the NMR timescale at room temperature. Thus, the total molar ratio of two conformers $[\boldsymbol{I}]_{\text{t}}/[\boldsymbol{V}]_{\text{t}}$ can be obtained by integration at certain $[\boldsymbol{G}]_{\text{t}}$. the relationship between $[\boldsymbol{I}]_{\text{t}}/[\boldsymbol{V}]_{\text{t}}$ and $[\boldsymbol{G}]$ can also be easily derived as (22).

$$\frac{[\boldsymbol{I}]_{\text{t}}}{[\boldsymbol{V}]_{\text{t}}}=\frac{[\boldsymbol{I}]+K_1[\boldsymbol{I}][\boldsymbol{G}]}{[\boldsymbol{V}]+K_2[\boldsymbol{V}][\boldsymbol{G}]}=\frac{[\boldsymbol{I}]+K_1[\boldsymbol{I}][\boldsymbol{G}]}{K[\mathbf{I}]+K_2K[\boldsymbol{I}][\boldsymbol{G}]}=\frac{1+K_1[\boldsymbol{G}]}{K(1+K_2[\boldsymbol{G}])} \quad (22)$$

By combining (16) with (22) to afford a theoretical expression of $[\boldsymbol{I}]_{\text{t}}/[\boldsymbol{V}]_{\text{t}}$ as the function of $[\boldsymbol{G}]_{\text{t}}$:

$$\frac{[\boldsymbol{I}]_\mathrm{t}}{[\boldsymbol{V}]_\mathrm{t}}=\frac{1+K_1\dfrac{-\left(1+K+(K_1+KK_2)([\boldsymbol{H}]_\mathrm{t}-[\boldsymbol{G}]_\mathrm{t})\right)+\sqrt{\left(1+K+(K_1+KK_2)([\boldsymbol{H}]_\mathrm{t}-[\boldsymbol{G}]_\mathrm{t})\right)^2+4(K_1+KK_2)(1+K)[\boldsymbol{G}]_\mathrm{t}}}{2(K_1+KK_2)}}{K(1+K_2\dfrac{-\left(1+K+(K_1+KK_2)([\boldsymbol{H}]_\mathrm{t}-[\boldsymbol{G}]_\mathrm{t})\right)+\sqrt{\left(1+K+(K_1+KK_2)([\boldsymbol{H}]_\mathrm{t}-[\boldsymbol{G}]_\mathrm{t})\right)^2+4(K_1+KK_2)(1+K)[\boldsymbol{G}]_\mathrm{t}}}{2(K_1+KK_2)})} \quad (23)$$

Modifying (23) gives us (24):

$$\frac{[\boldsymbol{I}]_\mathrm{t}}{[\boldsymbol{V}]_\mathrm{t}}=\frac{\dfrac{2(K_1+KK_2)+K_1\left\{-\left(1+K+(K_1+KK_2)([\boldsymbol{H}]_\mathrm{t}-[\boldsymbol{G}]_\mathrm{t})\right)+\sqrt{\left(1+K+(K_1+KK_2)([\boldsymbol{H}]_\mathrm{t}-[\boldsymbol{G}]_\mathrm{t})\right)^2+4(K_1+KK_2)(1+K)[\boldsymbol{G}]_\mathrm{t}}\right\}}{2(K_1+KK_2)}}{\dfrac{2K(K_1+KK_2)+KK_2\left\{-\left(1+K+(K_1+KK_2)([\boldsymbol{H}]_\mathrm{t}-[\boldsymbol{G}]_\mathrm{t})\right)+\sqrt{\left(1+K+(K_1+KK_2)([\boldsymbol{H}]_\mathrm{t}-[\boldsymbol{G}]_\mathrm{t})\right)^2+4(K_1+KK_2)(1+K)[\boldsymbol{G}]_\mathrm{t}}\right\}}{2(K_1+KK_2)}} \quad (24)$$

Then the final theoretical expression of $[\boldsymbol{I}]_\mathrm{t}/[\boldsymbol{V}]_\mathrm{t}$ with $[\boldsymbol{G}]_\mathrm{t}$ (25) can be get by reducing fraction of (24):

$$\frac{[\boldsymbol{I}]_\mathrm{t}}{[\boldsymbol{V}]_\mathrm{t}}=\frac{2(K_1+KK_2)+K_1\left\{-\left(1+K+(K_1+KK_2)([\boldsymbol{H}]_\mathrm{t}-[\boldsymbol{G}]_\mathrm{t})\right)+\sqrt{\left(1+K+(K_1+KK_2)([\boldsymbol{H}]_\mathrm{t}-[\boldsymbol{G}]_\mathrm{t})\right)^2+4(K_1+KK_2)(1+K)[\boldsymbol{G}]_\mathrm{t}}\right\}}{2K(K_1+KK_2)+KK_2\left\{-\left(1+K+(K_1+KK_2)([\boldsymbol{H}]_\mathrm{t}-[\boldsymbol{G}]_\mathrm{t})\right)+\sqrt{\left(1+K+(K_1+KK_2)([\boldsymbol{H}]_\mathrm{t}-[\boldsymbol{G}]_\mathrm{t})\right)^2+4(K_1+KK_2)(1+K)[\boldsymbol{G}]_\mathrm{t}}\right\}} \quad (25)$$

These equations (20), (21), and (25) are used to calculate the binding constants of the same guest to the two conformers.

3. Supplementary Methods

3.1 General method

All the reagents involved in this research were commercially available and used without further purification unless otherwise noted. Thin-layer chromatography (TLC) was carried out on 0.25 mm Yantai silica gel plates (60F-254). Column chromatography was performed on silica gel 60 (Tsingdao 40 – 63 nm, 200 – 300 mesh). ^{1}H, ^{13}C NMR, ^{1}H-^{1}H COSY and ^{1}H-^{1}H ROESY NMR spectra were recorded on Bruker Avance-400 (500, 600) spectrometers. All chemical shifts are reported in ppm with residual solvents or TMS (tetramethylsilane) as the internal standards. The following abbreviations were used for signal multiplicities: s, singlet; d, doublet; t triplet; m, multiplet. Electrospray-ionization time-of-flight high-resolution mass spectrometry (ESI-TOF-HRMS) experiments were conducted on an applied Q EXACTIVE mass spectrometry system. Compounds **S1** and **S4** were synthesized by following the literature procedures[3]. Absorption spectra were recorded on a Hitachi U-2600 UV−vis spectrophotometer. Stopped-flow experiments were performed with a SX20 system from Applied Photophysics. The samples were thermo stated at 25.0 ^{o}C for 5 min and were then mixed in a 1:1 ratio. The concentrations stated for the stopped-flow experiments are final concentrations and correspond to half of the concentrations of chemicals in each syringe. At least 5 individual kinetic traces were averaged. The kinetic data were analysed as single kinetics by fitting the data to a single-exponential function.

Quantum chemistry calculations: 3D structures of the five representative conformations were optimized at wB97XD/6-31G(d) level. Initially, five representative conformations **I-V** with different possible orientation of the naphthalene flipping for the host were built manually and then optimized by PM3 level of theory by using Spartan ’14 (Wavefunction, Inc.). Finally, single-point energy calculations on these structures were carried out at wB97XD/6-31G(d) level (solvent: CH_2Cl_2) by Gaussian 09 software package.[4] 3D structures of **2@1-I** and **2@1-V** were

optimized by the functional DFT (wB97XD/6-31G(d)) basis set in CH_2Cl_2.

3.2 Synthetic Procedures

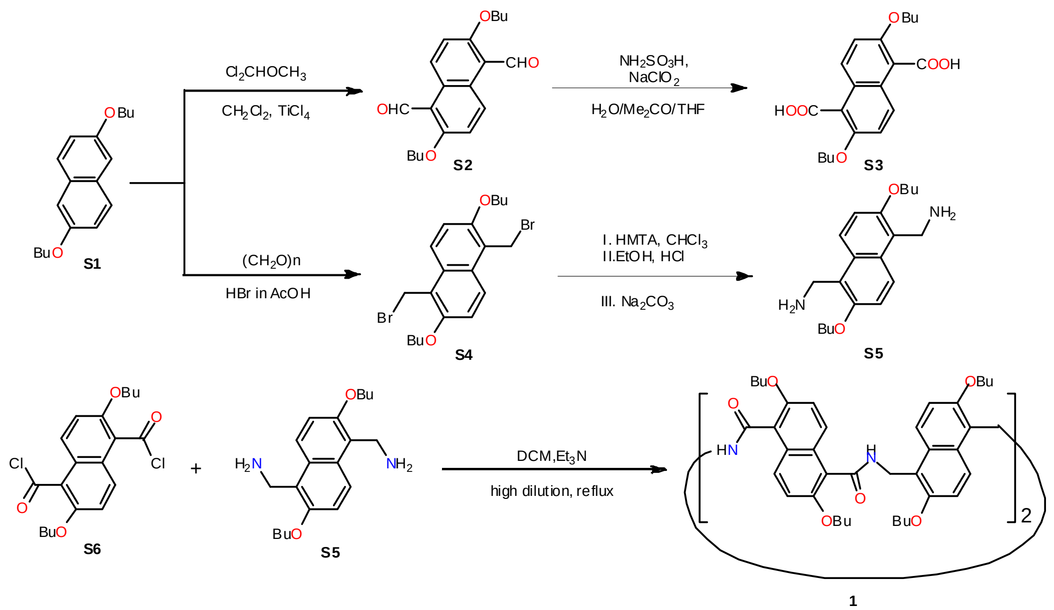

Supplementary Figure 42 Synthetic procedure of **1**

Synthesis of S2: To the solution of compound **S1** (10.0 g, 37 mmol) in dry CH_2Cl_2 (210 mL) was added 1,1-dichlorodimethyl ether (14.4 mL, 158.9 mmol, 4 equiv.) at 0 ^{o}C. Then $TiCl_4$ (16 mL, 147 mmol) was added slowly via a syringe. After stirring at 0 ^{o}C for 1 h, the resulting mixture was warmed to room temperature and stirred for another 3 h. The solution was poured into saturated aqueous solution of $NaHCO_3$ (400 mL). The mixture was then extracted with CH_2Cl_2 (200 mL × 3). The combined organic layers were washed with saturated $NaHCO_3$ (200 mL) and H_2O (200 mL), and then dried over anhydrous Na_2SO_4. The solvent was removed with rotary evaporator to give the crude product which was purified by column chromatography (SiO_2, hexane) to afford pure dialdehyde **S2** (9.5 g, 79%) as a yellow solid. mp = 171-172 ˚C; ^{1}H NMR (500 MHz, $CDCl_3$): δ 10.81 (s, 2H), 9.46 (d, J = 9.6 Hz, 2H), 7.31 (d, J = 9.6 Hz, 2H), 4.15 (t, J = 6.4 Hz, 4H), 1.82 – 1.76 (m, 4H), 1.52 – 1.43 (m, 4H), 0.93 (t, J = 7.4 Hz, 6H); ^{13}C NMR (126 MHz, $CDCl_3$): δ 192.33, 162.24, 134.37, 126.45, 116.93, 116.32, 69.24, 31.31, 19.29, 13.83; HRMS (m/z): $[M+H]^+$ calcd for $C_{20}H_5O_4^+$, 329.17474; found, 329.17447 (error = -0.8 ppm).

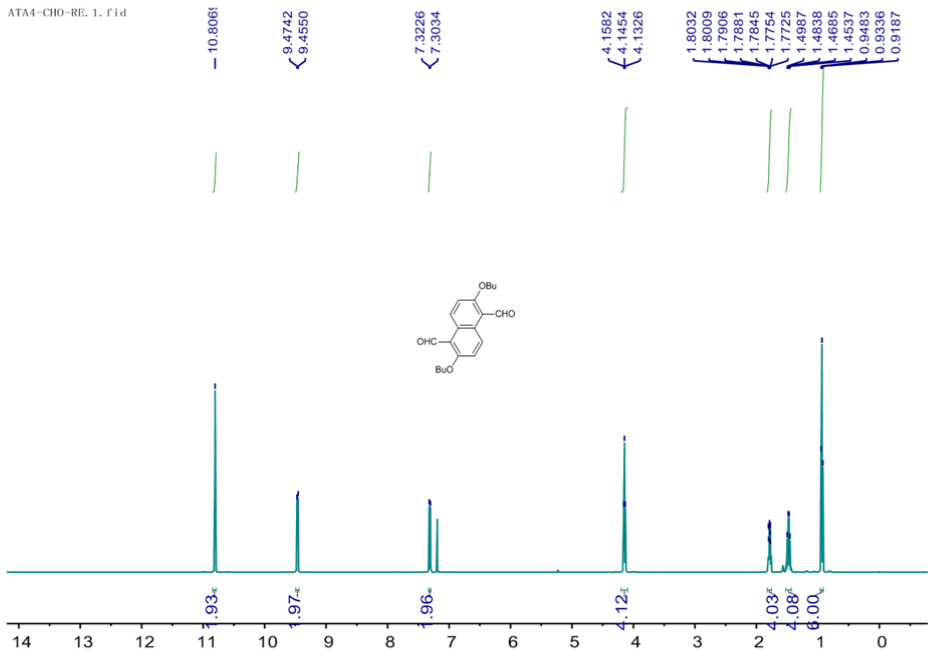

Supplementary Figure 43 ^{1}H NMR spectrum (500 MHz, $CDCl_3$, 25 ^{o}C) of **S2**

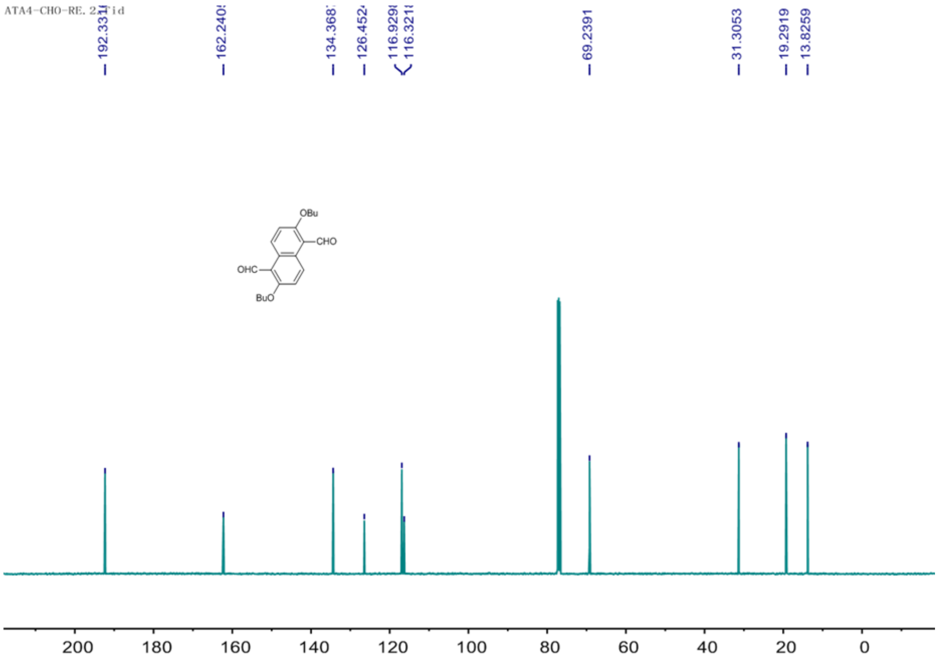

Supplementary Figure 44 ^{13}C NMR spectrum (126 MHz, $CDCl_3$, 25 ^{o}C) of **S2**

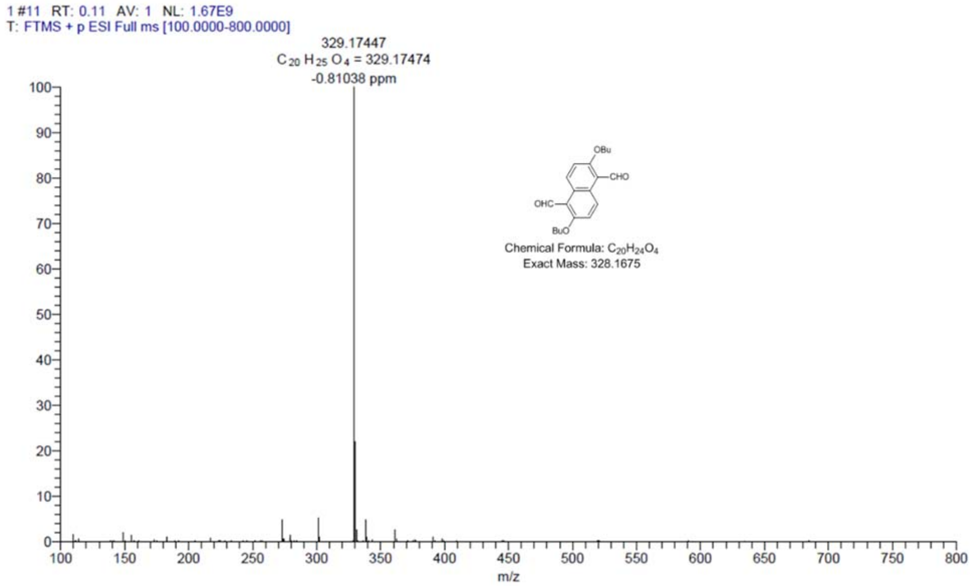

Supplementary Figure 45 ESI mass spectrum of **S2**

Synthesis of S3: Dialdehyde **S2** (1.0 g, 3.1 mmol) was dissolved in the mixture of acetone (35 mL), THF (35 mL), and H_2O (70 mL) and cooled to 0°C. To this solution, sulfamic acid (1.8 g, 18.3 mmol) and sodium chlorite (1.7 g, 18.3 mmol, 80%) were slowly added. The resulting mixture was warmed to room temperature and stirred overnight. The mixture was concentrated under reduced pressure to remove the organic solvent. The aqueous solution was acidified with 6 M HCl. The precipitate was collected through filtration and dried to afford diacid **S3** (0.77 g, 70%) as a white solid. mp = 208-210˚C; ^{1}H NMR (500 MHz, DMSO-d_6): δ 13.24 (s, 2H), 7.72 (d, J = 9.2 Hz, 2H), 7.53 (d, J = 9.4 Hz, 2H), 4.14 (t, J = 6.3 Hz, 4H), 1.69 (m, 4H), 1.50 – 1.42 (m, 4H), 0.93 (t, J = 7.4 Hz, 6H); ^{13}C NMR (126 MHz, DMSO-d_6): δ 168.83, 151.51, 126.63, 125.32, 120.11, 117.23, 69.29, 31.44, 19.06, 14.16; HRMS (m/z): [M-H]$^-$ $C_{20}H_{23}O_6^-$, 359.15001, found 359.15024 (error = +0.6 ppm).

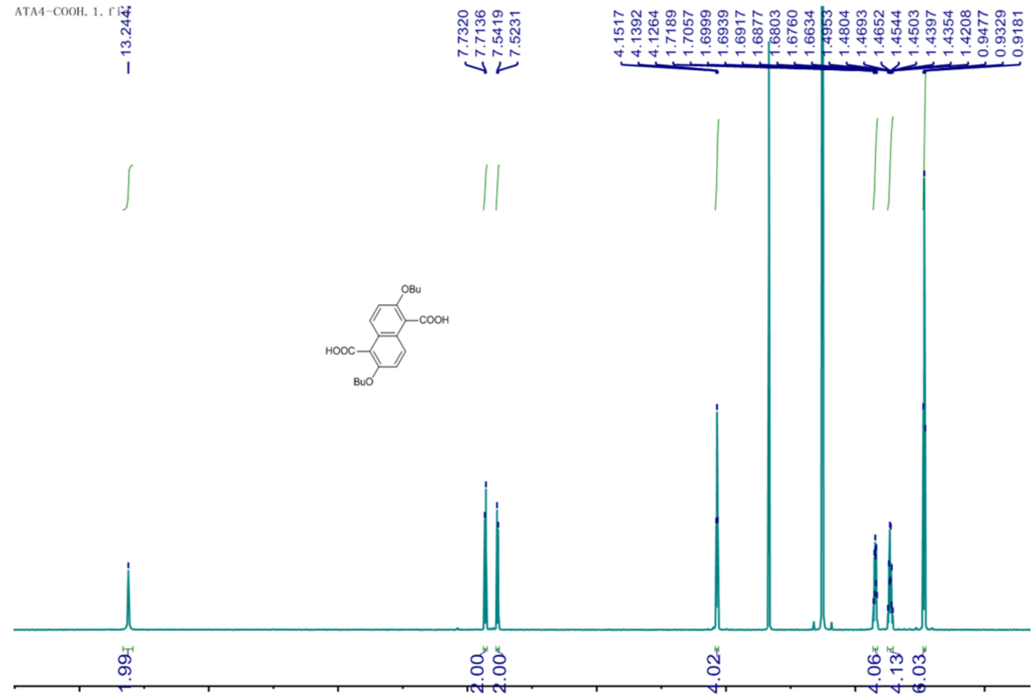

Supplementary Figure 46 ^{1}H NMR spectrum (500 MHz, DMSO-d_6, 25 ^{o}C) of **S3**

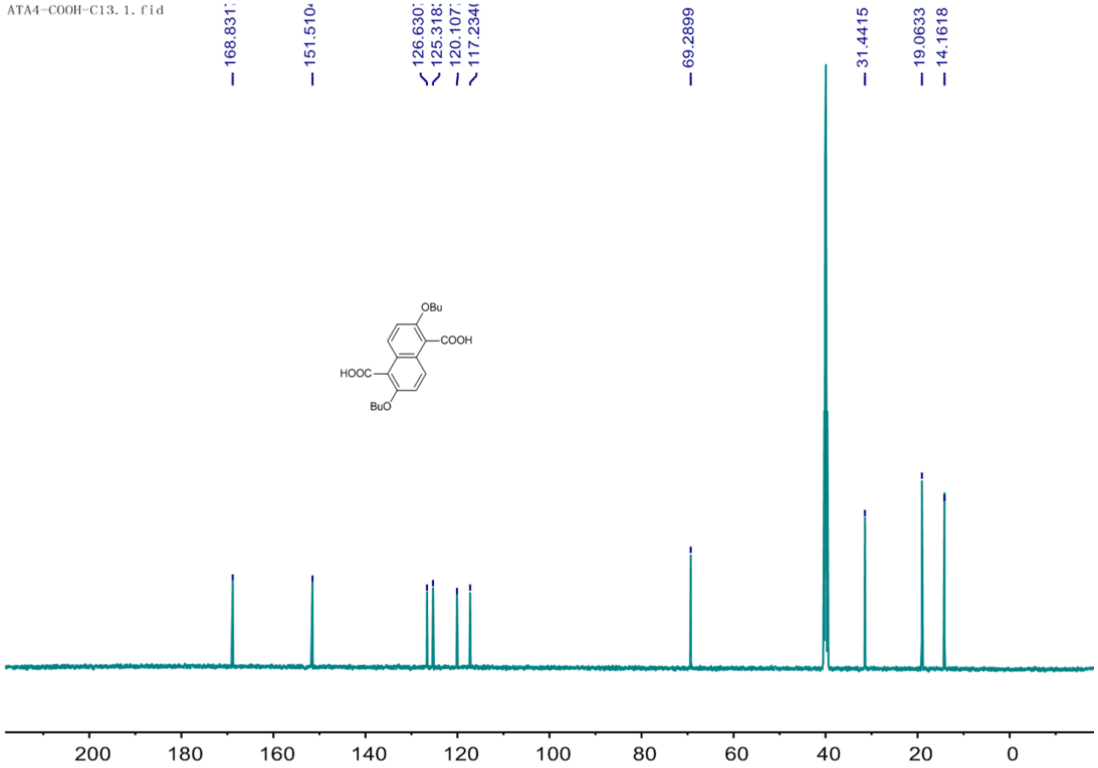

Supplementary Figure 47 ^{13}C NMR spectrum (126 MHz, DMSO-d_6, 25 ^{o}C) of **S3**

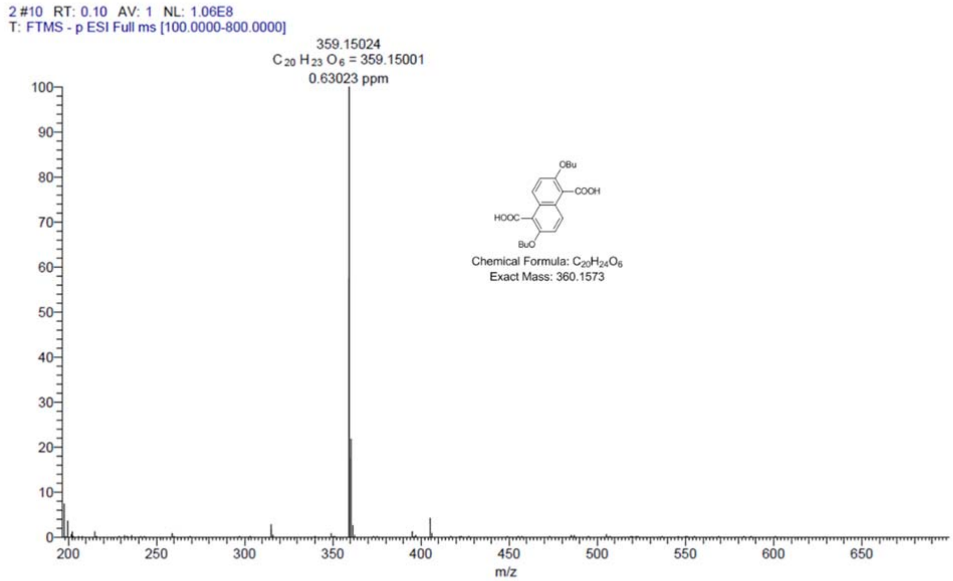

Supplementary Figure 48 ESI mass spectrum of **S3**

Synthesis of S5: A mixture of S**4** (0.53 g, 1.15 mmol) and hexamethylenetetramine (0.49 g, 3.46 mmol) in chloroform (50 mL) was refluxed for 48 h. After cooling to room temperature, the precipitate was collected by filtration. The solid was then dissolved in the mixture of ethanol (50 mL) and HCl (30%, 20 mL). The solution was stirred at 80 ^{o}C for 48 h. After cooled to 0 ^{o}C, the resulting precipitate was collected by filtration and washed with cold ethanol. The solid was then suspended in NaOH aq. (1 M, 50 mL) with stirring. CH_2Cl_2 (100 mL) was added to extract the neutral diamine (**S5**). The aqueous phase was extracted with additional CH_2Cl_2 (100 mL). The organic phases were combined and dried over $MgSO_4$. The solvent was removed to give diamine **S5** (0.20 g, 50%) as a gray solid. mp = 123-125˚C; ^{1}H NMR (500 MHz, $CDCl_3$): δ 7.89 (d, J = 9.3 Hz, 2H), 7.22 (d, J = 9.3 Hz, 2H), 4.19 (s, 4H), 4.03 (t, J = 6.4 Hz, 4H), 1.74 (dd, J = 8.6, 6.3 Hz, 4H), 1.53 – 1.45 (m, 4H), 0.92 (t, J = 7.4 Hz, 6H); ^{13}C NMR (126 MHz, $CDCl_3$): δ 152.11, 128.16, 125.39, 123.19, 115.57, 69.10, 36.47, 31.77, 19.44, 13.93; HRMS (m/z): $[M+Na]^+$, $C_{20}H_{30}O_2N_2Na^+$, 353.21995, found 353.21951 (error = -1.2 ppm).

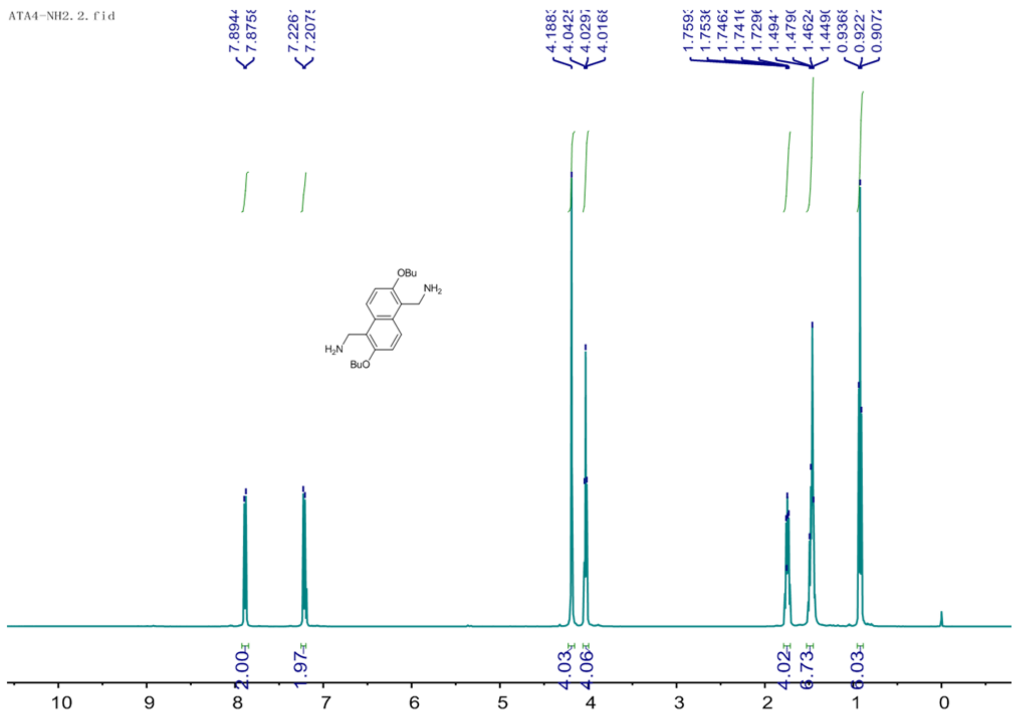

Supplementary Figure 49 ^{1}H NMR spectrum (500 MHz, $CDCl_3$, 25 °C) of **S5**

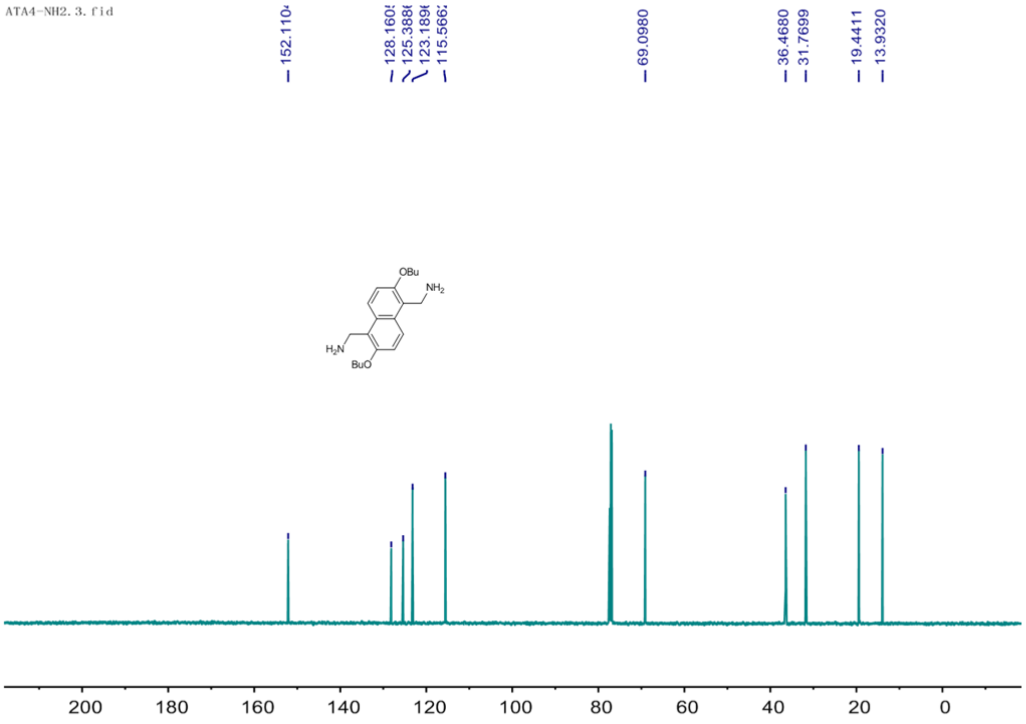

Supplementary Figure 50 ^{13}C NMR spectrum (126 MHz, $CDCl_3$, 25 °C) of **S5**

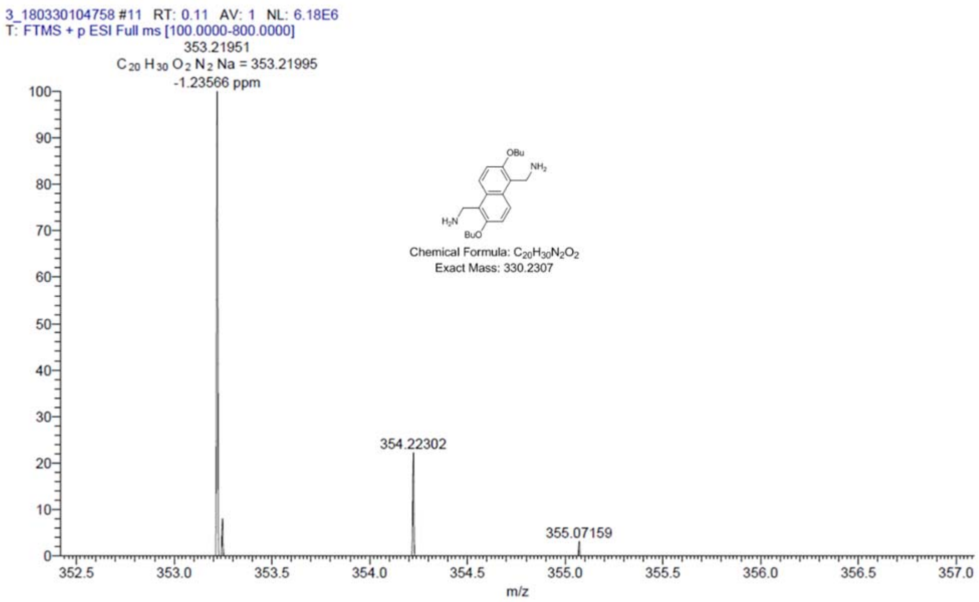

Supplementary Figure 51 ESI mass spectrum of compound **S5**

Synthesis of 1: Firstly, oxalyl chloride (4.3 mL, 52 mmol) was added to the solution of Diacid **S3** (3.1 g, 8.6 mmol) in dry CH_2Cl_2 (100 mL) at room temperature. The reaction mixtures were stirred for 30 min and DMF (1 drops) was added. The mixtures were stirred overnight and then evaporated to dryness. Compound **S6** (3.2 g, 95%) was obtained as lightly yellow solid. The residues were used directly in the synthesis of **1**.

To the mixture of dry TEA (16 mL) in dry CH_2Cl_2 (500 mL) at room temperature was added the solution of **S6** (2.27 g, 5.7 mmol) and **S5** (1.89 g, 5.7 mmol) in dry CH_2Cl_2 (60 mL) dropwise through a syringe pump during 20 h under nitrogen. The mixture was stirred overnight and the solvent was removed to dryness under reduced pressure. The residue was dissolved in CH_2Cl_2 (200 mL) and washed with saturated aqueous NH_4Cl (100 mL) and dried over Na_2SO_4. Then, the solvent was removed with rotary evaporator to give the crude product which was purified by column chromatography (SiO_2, CH_2Cl_2 /methanol =100: 2) to afford pure **1** as a white solid (530 mg, 14%). mp > 300˚C; ^{1}H NMR (500 MHz, CD_2Cl_2): δ 8.14 – 8.08 (m, 4H), 7.91 – 7.86 (m, 4H), 7.34 – 7.30 (m, 4H), 7.04– 7.01 (m, 4H), 5.54 – 5.44 (m, 2H), 5.39 (s, 2H), 5.29

(d, J = 8.4 Hz, 4H), 5.02 – 4.90 (m, 4H), 4.15 – 4.01 (m, 8H), 4.00 – 3.83 (m, 8H), 1.88 – 1.77 (m, 8H), 1.64 (q, J = 7.0 Hz, 8H), 1.58 – 1.49 (m, 8H), 1.42 (dt, J = 15.6, 7.8 Hz, 8H), 1.08 – 0.82 (m, 24H); ^{13}C NMR (126 MHz, CD_2Cl_2): δ 165.86, 165.78, 153.19, 153.07, 151.47, 151.37, 128.06, 128.02, 127.29, 126.95, 126.48, 126.28, 124.99, 121.34, 120.96, 119.76, 119.56, 116.02, 115.95, 115.37, 69.64, 69.55, 69.26, 69.16, 33.78, 33.66, 31.66, 31.64, 31.37, 31.34, 29.69, 19.34, 19.08, 19.05, 13.68, 13.62, 13.60; HRMS (m/z): $[M+H]^+$ $C_{80}H_{101}O_4N_4^+$, 1309.74105, found 1309.74377 (error = 2.1 ppm).

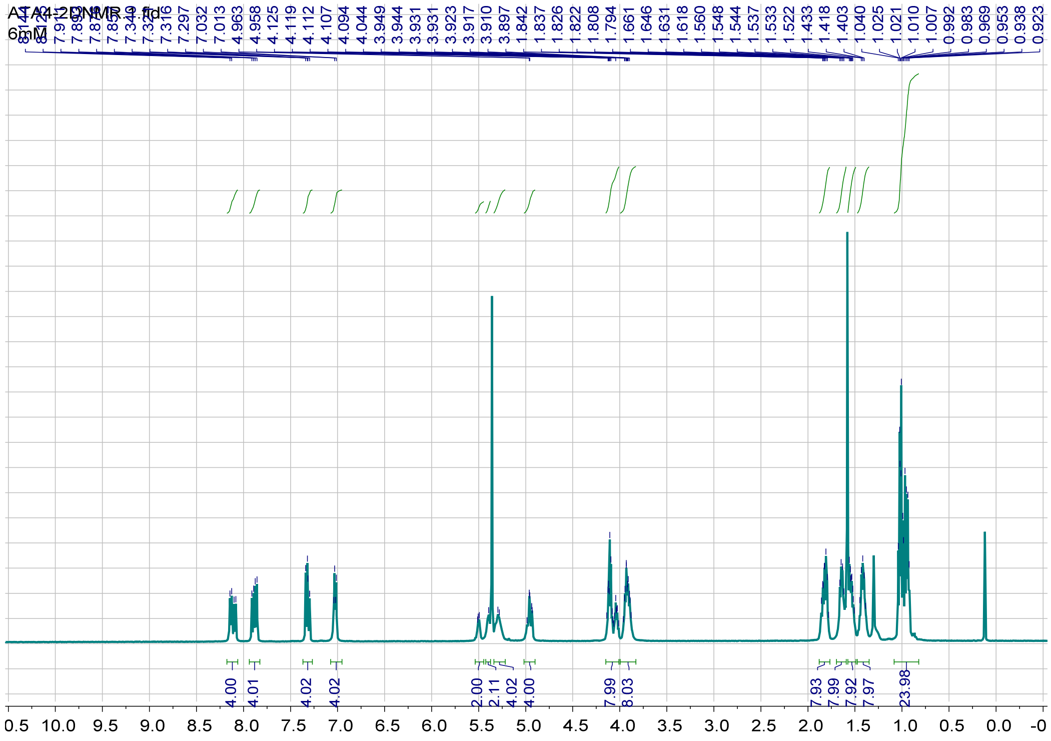

Supplementary Figure 52 ^{1}H NMR spectrum (500 MHz, CD_2Cl_2, 25 ^{o}C) of **1**

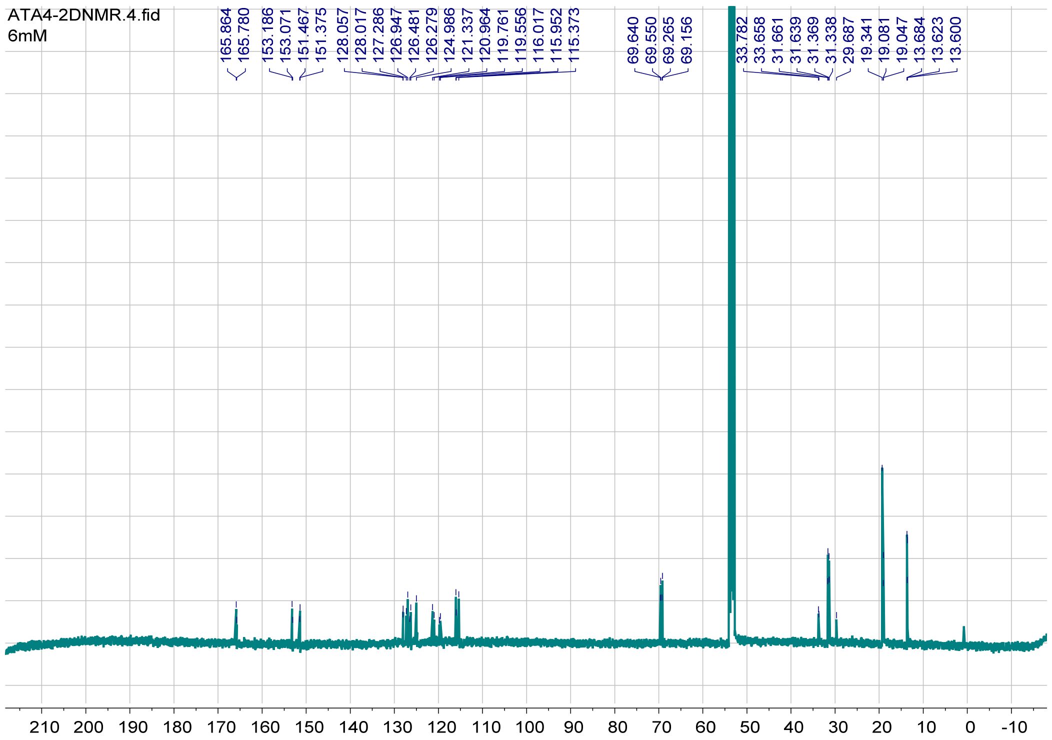

Supplementary Figure 53 ^{13}C NMR spectrum (126 MHz, CD_2Cl_2, 25 ^{o}C) of **1**

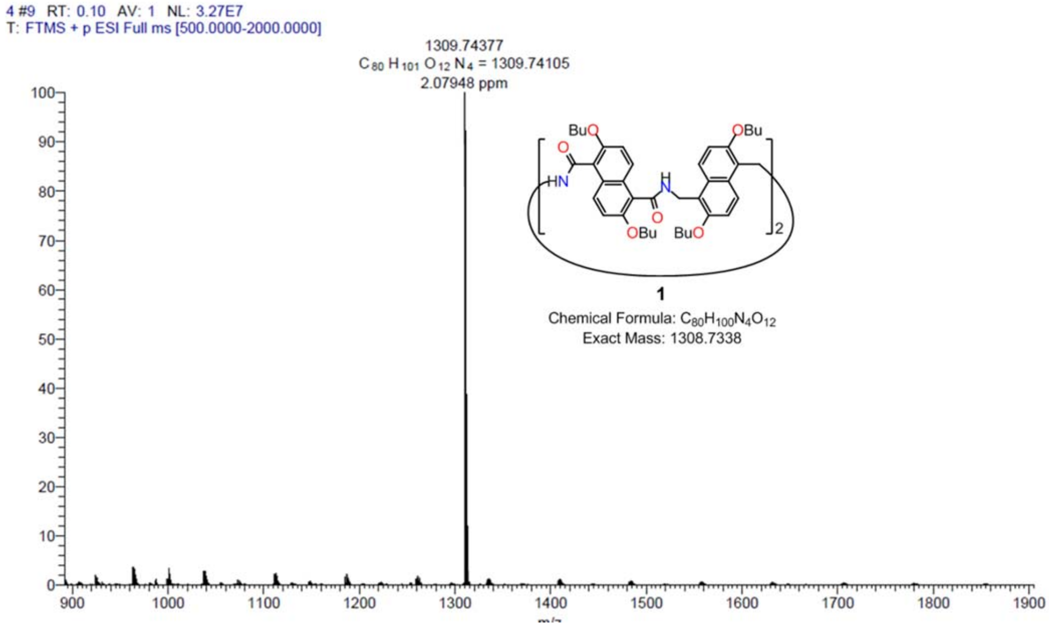

Supplementary Figure 54 ESI mass spectrum of compound **1**

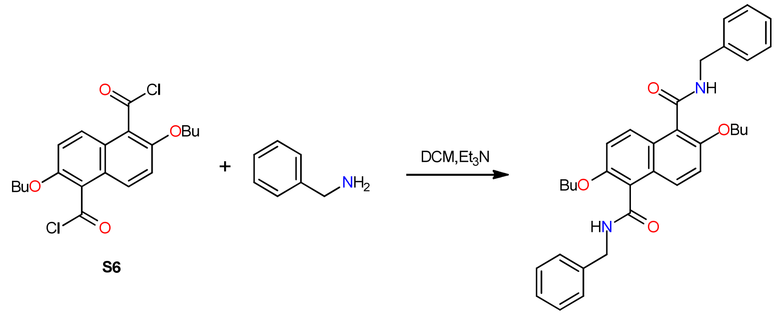

Supplementary Figure 55 Synthesis of an acyclic amide compound (S7)

The mixture of **S6** (200 mg, 0.5 mmol), benzylamine (216 mg, 2.0 mmol) and TEA (204 mg, 2.0 mmol) in dry CH_2Cl_2 (5 mL) were stirred at room temperature for 24 h. The precipitate was collected through filtration and washed with CH_2Cl_2 (10 mL), affording the acyclic amide compound **S7** (210 mg, 77%). mp = 245-246˚C; ^{1}H NMR (500 MHz, CD_2Cl_2): δ 8.07 (d, *J* = 9.3 Hz, 2H), 7.51 – 7.46 (m, 4H), 7.42 – 7.39 (m, 4H), 7.37 – 7.31 (m, 4H), 6.48 (t, *J* = 6.0 Hz, 2H), 4.75 (d, *J* = 6.0 Hz, 4H), 4.14 (t, *J* = 6.6 Hz, 4H), 1.80 – 1.73 (m, 4H), 1.51 – 1.43 (m, 4H), 0.98 (t, *J* = 7.4 Hz, 6H); ^{13}C NMR (101 MHz, CF_3COOD): δ 171.48, 154.34, 129.74, 128.92, 128.69, 128.51, 128.31, 127.88, 125.03, 118.24, 69.60, 46.85, 45.03, 30.32, 18.11; HRMS (m/z): $[M+H]^+$ $C_{34}H_{39}N_2O_4^+$, 539.2904, found 539.2902 (error = -0.4 ppm).

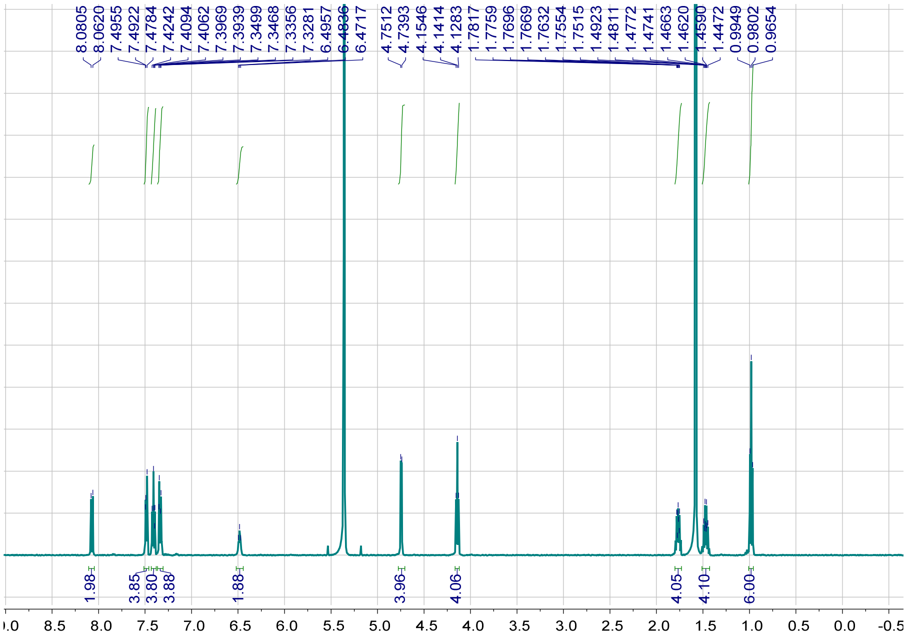

Supplementary Figure 56 ^{1}H NMR spectrum (500 MHz, CD_2Cl_2, 25 °C) of **S7**

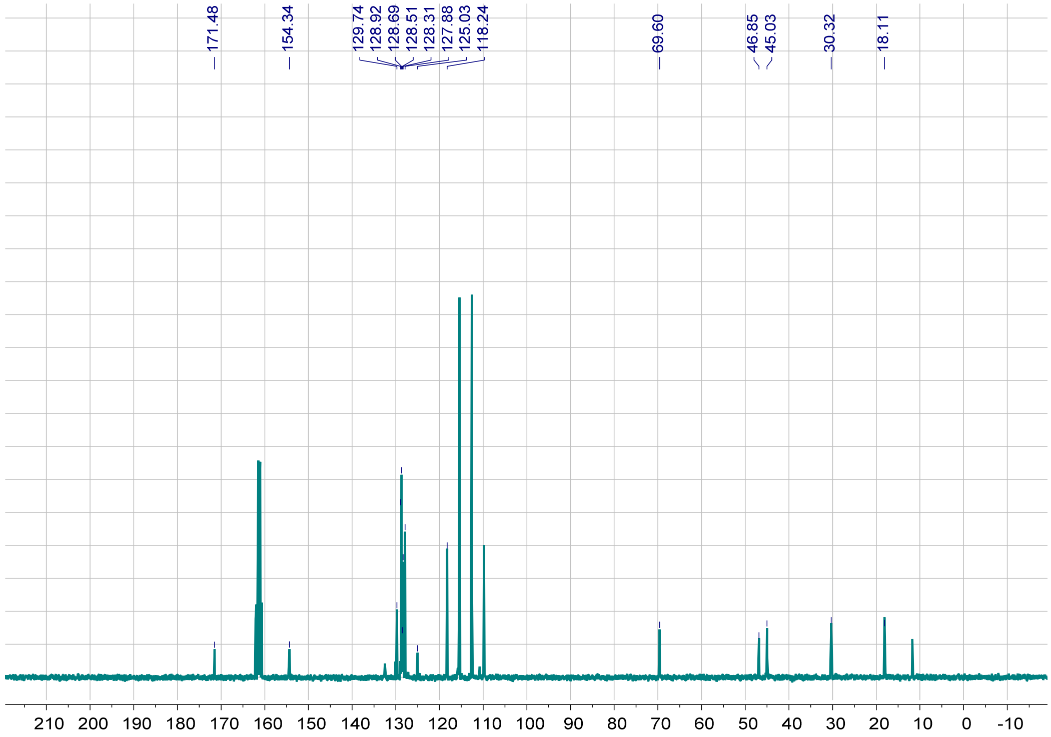

Supplementary Figure 57 ^{13}C NMR spectrum (101 MHz, CF_3COOD, 25 ^oC) of **S7**

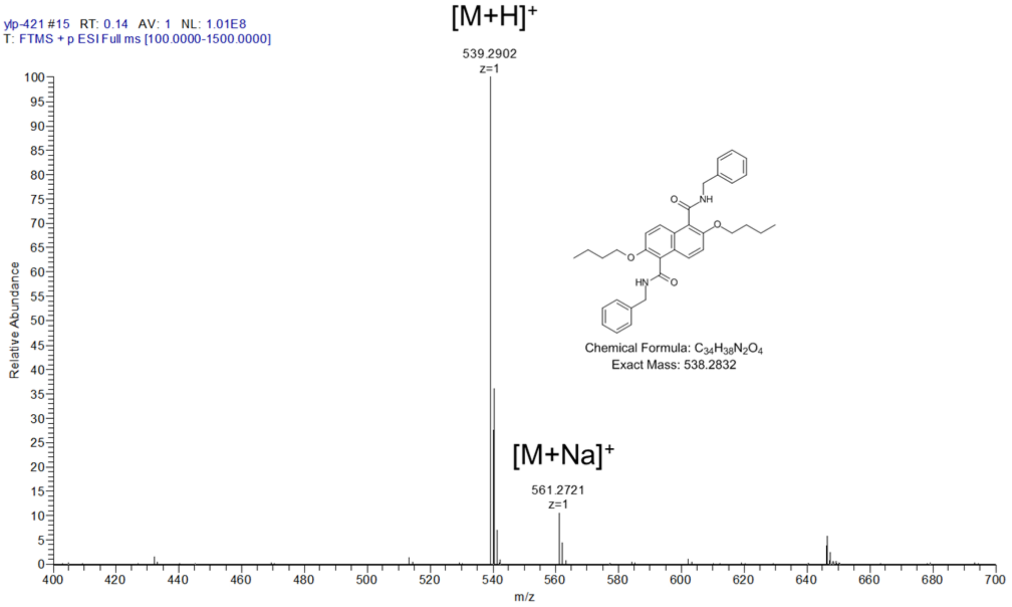

Supplementary Figure 58 ESI mass spectrum of **S7**

3.3 X-ray Crystallography

Single crystal X-ray data in the present study were collected at 120 K on an Agilent Supernova (dual source) diffractometer with Atlas detector and mirror monochromatized Cu-Kα (λ = 1.54184 Å) radiation. The CrysAlisPro[5] software was used for data collection (θ and ω scans) and processing. The structures were solved by intrinsic phasing methods (SHELXT[6]) and refined by full-matrix least squares on F^2 using SHELXL[7] in the OLEX2 program package.[8] The crystals were all very weakly diffracting, even with extremely prolonged exposure times using Cu radiation, with no reflections observed beyond 1.0 Å resolution. This is routinely observed for supramolecular structures, especially when they are comprised of predominantly light atoms as with **2@1**, so data was collected to as high a resolution as possible, with the identity of the structure being clearly established without issue. Anisotropic displacement parameters were assigned to non-H atoms. Positional disorder in the structures was treated by gently restraining geometric and anisotropic displacement parameters. All hydrogen atoms were refined using riding models with U_{eq}(H) of 1.5U_{eq}(C) for terminal methyl groups and of 1.2U_{eq}(N) for the amide groups. The restraints DFIX and DANG were used to ensure the chemical sensibility (bond lengths and angles) of the disordered ^{n}butyl substituents. The restraints DELU, SIMU, RIGU, and in two instances the EADP constraint, were used to modify the thermal parameters of the structure, which were required due to the disordered ^{n}butyl substituents, but also to account for the light atom nature of the structure and the low resolution of the data collected as a result. The main details of crystal data collection and refinement parameters are presented below. CCDC 1950443 (**2@1**) contains the supplementary crystallographic data for this paper. These data can be obtained free of charge via http://www.ccdc.cam.ac.uk/conts/retrieving.html (or from the CCDC, 12 Union Road, Cambridge CB2 1EZ, UK; Fax: +44 1223 336033; E-mail: deposit@ccdc.cam.ac.uk).

Structure of 2@1: Compound **2@1** was crystallized as red plates with vapour diffusion of pentane into a CH_2Cl_2 solution of an equimolar mixture of the host (macrocycle **1**) and guest (guest **2**). Crystal data and refinement parameters of **2@1**: $C_6H_4N_2O_4@C_{80}H_{100}N_4O_{12}$, M = 1477.75, monoclinic, space group $P2_1/c$, a = 17.267(3), b = 24.991(2), c = 19.087(4) Å, β = 111.20(2), V = 7679(2) $Å^3$, Z = 4, ρ_{calc} = 1.278 Mgm^{-3}, μ = 0.71 mm^{-1}, F000 = 3160, θ range = 2.7-49.9°, 16273 reflections collected of which 8368 unique (R_{int} = 0.088), No. of reflections with $I>2\sigma(I)$ = 3941, which were used in all calculations, 1058 parameters and 528 restraints, Goodness-of-fit (F^2) = 1.05. The final R indices [$I>2\sigma(I)$]: R_1 = 0.173 and wR_2 = 0.412. R indices (all data): R_1 = 0.264 and wR_2 = 0.475. Largest residual electron densities: 0.53 and -0.52 e $Å^{-3}$.

4. Supplementary References

1 . Ogoshi, T. et al. Synthesis and conformational characteristics of alkyl-Substituted pillar[5]arenes. *J. Org. Chem.* **75**, 3268–3273 (2010).

2 . (a) Thordarson, P. Determining association constants from titration experiments in supramolecular chemistry. *Chem. Soc. Rev.* **40**, 1305–1323 (2011); (b) Thordarson, P. *Binding Constants and Their Measurement*, (John Wiley & Sons, Hoboken, 2012).

3 . Jia, F. et al. Oxatub[4]arene: a smart macrocyclic receptor with multiple interconvertible cavities. *Chem. Sci.* **6**, 6731–6738 (2015).

4. M. J. Frisch, G. W. T., H. B. Schlegel, G. E. Scuseria, M. A. Robb, J. R. Cheeseman, G. Scalmani, V. Barone, B. Mennucci, G. A. Petersson, H. Nakatsuji, M. Caricato, X. Li, H. P. Hratchian, A. F. Izmaylov, J. Bloino, G. Zheng, J. L. Sonnenberg, M. Hada, M. Ehara, K. Toyota, R. Fukuda, J. Hasegawa, M. Ishida, T. Nakajima, Y. Honda, O. Kitao, H. Nakai, T. Vreven, J. A. Montgomery, Jr., J. E. Peralta, F. Ogliaro, M. Bearpark, J. J. Heyd, E. Brothers, K. N. Kudin, V. N. Staroverov, R. Kobayashi, J. Normand, K. Raghavachari, A. Rendell, J. C. Burant, S. S. Iyengar, J. Tomasi, M. Cossi, N. Rega, J. M. Millam, M. Klene, J. E. Knox, J. B. Cross, V. Bakken, C. Adamo, J. Jaramillo, R. Gomperts, R. E. Stratmann, O. Yazyev, A. J. Austin, R. Cammi, C. Pomelli, J. W. Ochterski, R. L. Martin, K. Morokuma, V. G. Zakrzewski, G. A. Voth, P. Salvador, J. J. Dannenberg, S. Dapprich, A. D. Daniels, Ö. Farkas, J. B. Foresman, J. V. Ortiz, J. Cioslowski, D. J. Fox. *Gaussian 09*, revision D.01; Gaussian, Inc. Wallingford, CT: 2009.

5. Agilent, 2014. CrysAlis PRO. Agilent Technologies Ltd, Yarnton, Oxfordshire, England.

6. Sheldrick, G. M. SHELXT-Integrated space-group and crystal-structure determination. *Acta Cryst. A71*, 3–8 (2015).

7. Sheldrick, G. M. crystal structure refinement with SHELXL. *Acta Cryst.* **C71**, 3–8 (2015).

8. Dolomanov, O. V., Bourhis, L. J., Gildea, R. J., Howard, J. A. K. & Puschmann, H. OLEX2: A complete structure solution, refinement and analysis program *J. Appl. Cryst.* **42**, 339–341 (2009).
